# Supplementary material for: Cryo-EM structure of MukBEF reveals DNA loop entrapment at chromosomal unloading sites
Source: Mol Cell. 2021 Dec 2;81(23):4891–4906.e8. doi: 10.1016/j.molcel.2021.10.011 (PMC8669397; doi:10.1016/j.molcel.2021.10.011)
Supplement: Document S1. Figures S1–S7 and Tables S1 and S2 [file mmc1.pdf]

Molecular Cell, Volume *81*

Supplemental information

Cryo-EM structure of MukBEF reveals DNA loop entrapment at chromosomal unloading sites

Frank Bürmann, Louise F.H. Funke, Jason W. Chin, and Jan Löwe

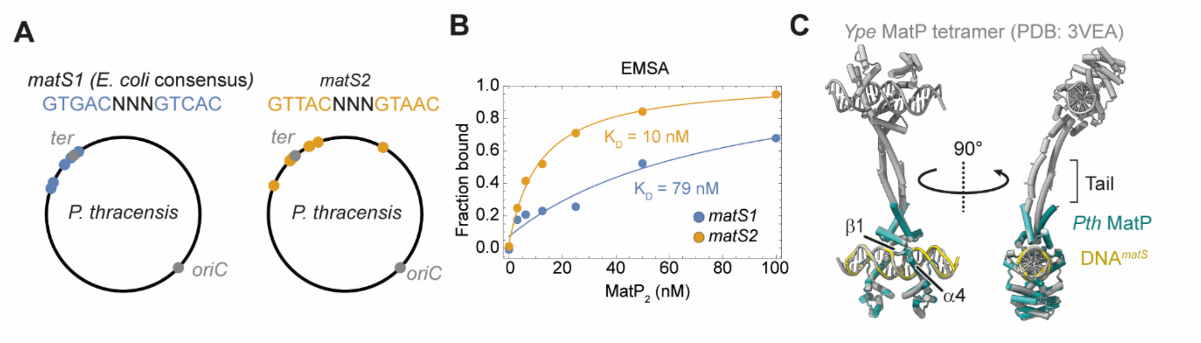

Figure S1. *matS* binding by *P. thracensis* MatP. Related to **Figure 1**.
(**A**) Location of the *E. coli matS* consensus sequence (left) and the *matS* sequence used for structure determination (right) mapped onto the *P. thracensis* chromosome. (**B**) Affinities of *P. thracensis* MatP for *matS* sites shown in A as determined by EMSA. (**C**) Superimposition of MatP–*matS* in the MukBEF-bound form (colored) and a crystal structure in the absence of MukBEF (gray, PDB: 3VEA). Positions of the *matS* binding elements $\alpha4$ and $\beta1$ and the C-terminal tetramerization tail are indicated. *Ype*, *Yersinia pestis*; *Pth*, *P. thracensis*.

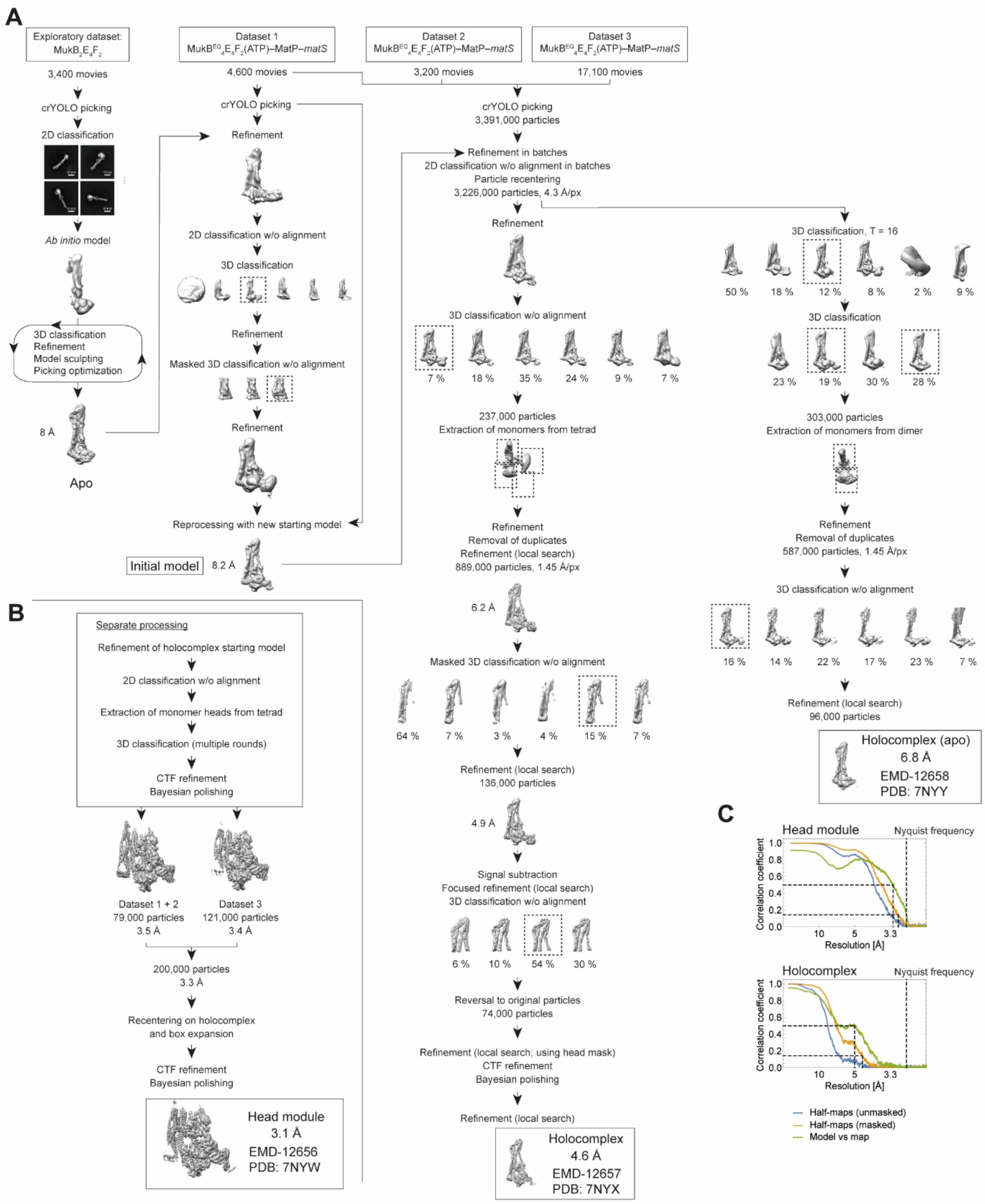

Figure S2. Cryo-EM data analysis workflow. Related to **Figures 1**-**3**.
(**A**) The processing tree for structure determination of MukBEF–MatP–DNA and apo-MukBEF monomers. (**B**) Data processing tree for focused structure determination of the head module. (**C**) FSC curves for MukBEF–MatP–DNA head module and holocomplex structures.

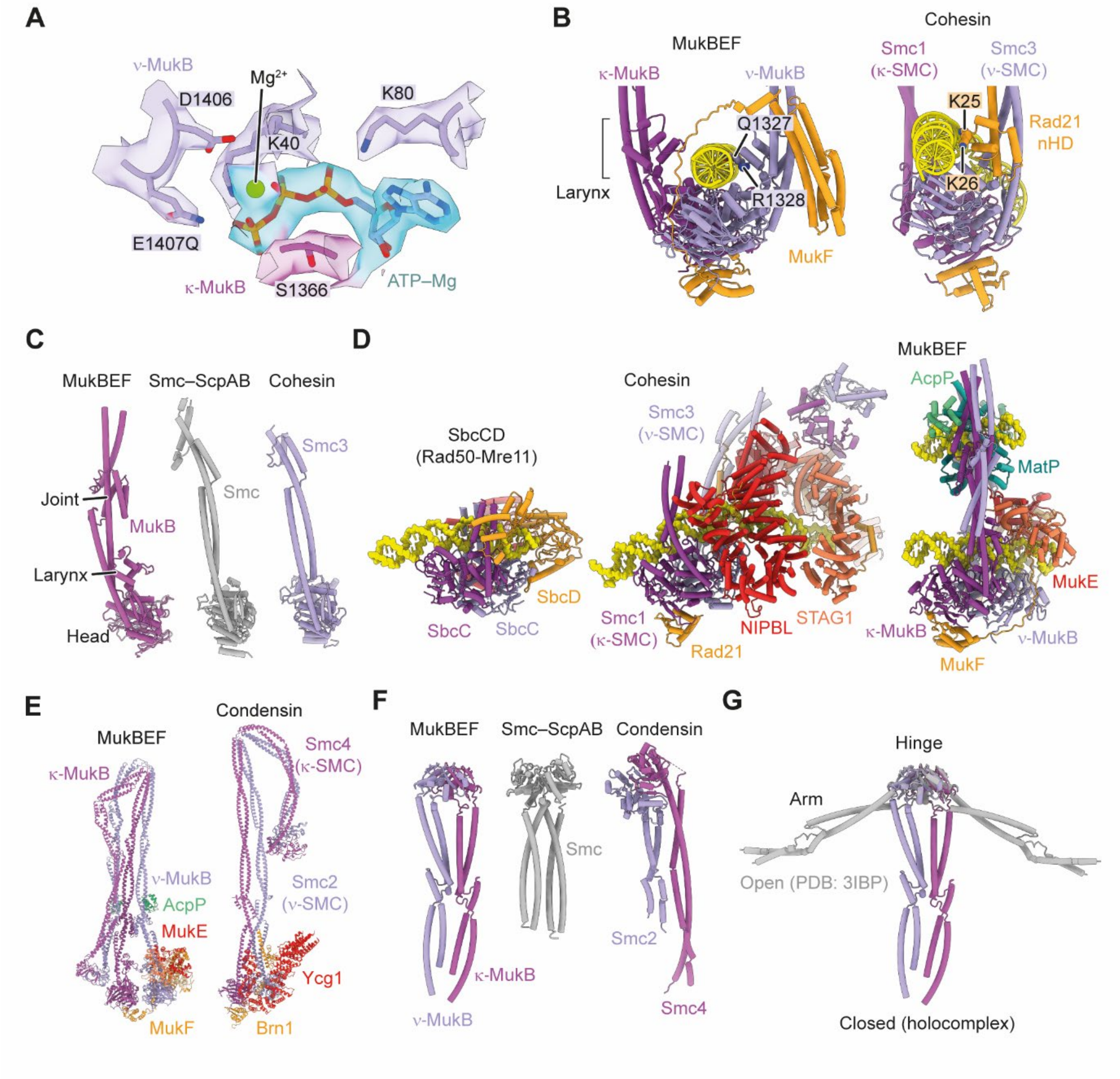

Figure S3. Conserved structural features and comparison with other SMC complexes. Related to **Figures 1**-**4**.

(**A**) Cryo-EM density and atomic model for the nucleotide binding site at the ν-MukB Walker A and B motives and the κ-MukB signature motive. (**B**) Architecture of the neck gate in MukBEF (left) and cohesin (right, PDB: 6WG3). An asymmetric DNA contact at the larynx of MukBEF and the N-terminal helical domain of Rad21 is indicated. (**C**) Comparison of the head-proximal regions of MukB, Smc (PDB: 5XEI) and Smc3 (PDB: 6WGE). (**D**) Architecture of SMC–DNA clamps. Models were aligned on the κ-SMC ATPase domain. PDB: 6S85, 6WG3.
(**E**) Comparison of apo-MukBEF and apo-condensin (PDB: 6YVU). (**F**) Comparison of the hinge-proximal regions of MukB, Smc (PDB: 4RSJ) and Smc2/4 (PDB: 4RSI).
(**G**) Superimposition of the hinge-proximal region of MukB in an open conformation (PDB: 3IBP) and in the closed conformation.

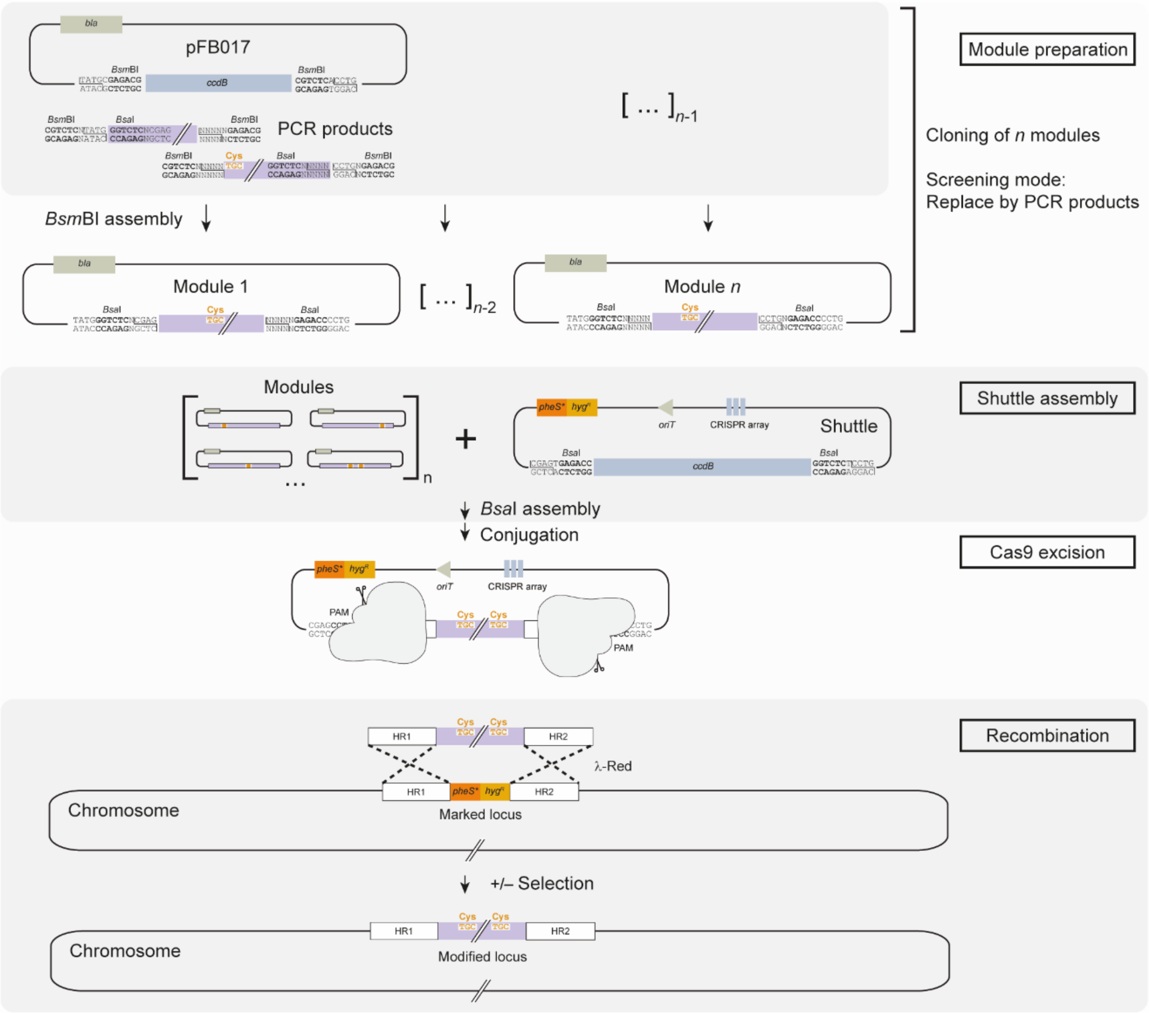

Figure S4. DNA assembly and excision scheme used for REXER-based strain construction. Related to **Figure 5**.

Modules containing cysteine point mutations were prepared as cloned and verified plasmids but can be substituted by linear PCR products (module preparation phase). Modules were assembled into a shuttle backbone (shuttle assembly phase), conjugated, and excised *in vivo* (Cas9 excision phase). Design of 30 bp homology crRNA for scarless Cas9 excision is indicated. The targeting construct is designed such that 50 bp homology regions (HR) direct the recombination into the target locus (recombination phase). Recombinants are selected for a positive marker in the targeting construct and against the *pheS** marker at the recipient locus.

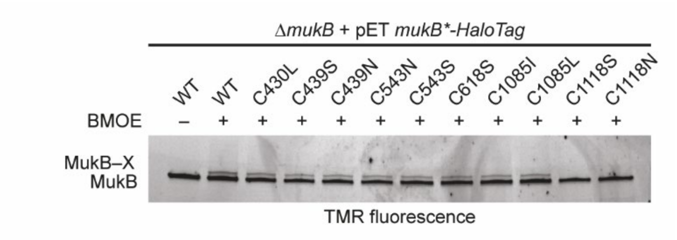

Figure S5. Background cross-linking of endogenous MukB cysteines. Related to **Figure 5**. Screening of endogenous cysteines for background cross-linking. *E. coli* Δ*mukB* was transformed with plasmids carrying *mukB-HaloTag* variants under control of a T7 promoter. Leaky expression in the strain lacking a T7 RNA polymerase gene produced about 40 **%** MukB-HaloTag compared to *mukB-HaloTag* expressed from the endogenous locus. The *mukB* null phenotype was complemented in all cases. Cells were treated with BMOE and proteins were detected by in-gel fluorescence. C1118S and C1118N abolished background cross-linking with a low-molecular weight protein.

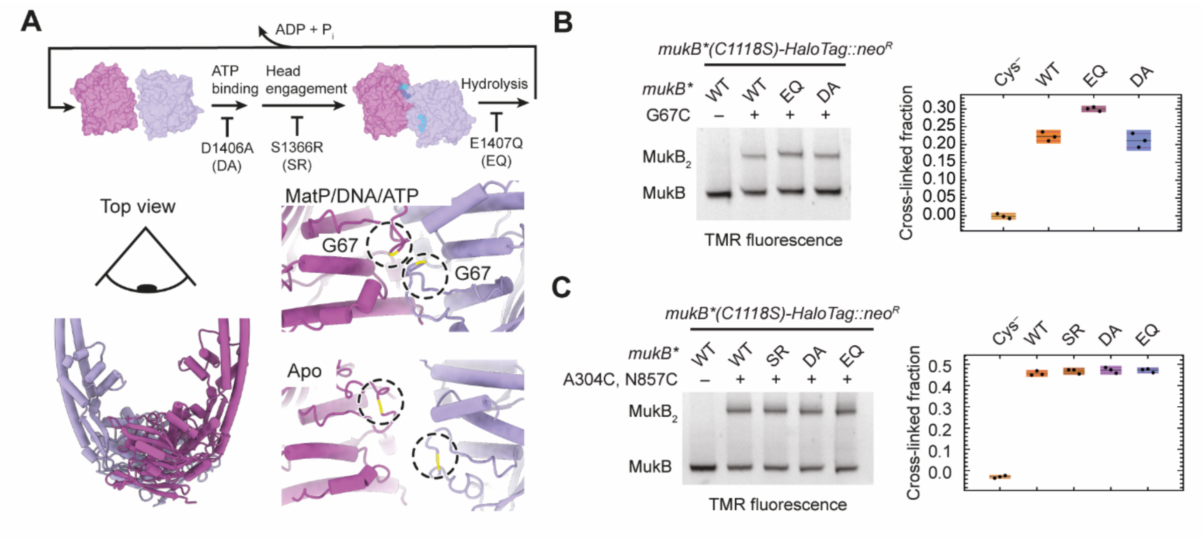

Figure S6. *In vivo* head engagement and arm folding in ATPase mutants. Related to **Figures 5** and **6**.

(**A**) Schematic of the ATP hydrolysis cycle and blocking mutations (top). Location of the head engagement sensor residue G67 in MatP/DNA/ATP and apo states (bottom). S1366R ('SR') blocks head engagement, D1406A ('DA') blocks ATP binding, E1407Q ('EQ') blocks ATP hydrolysis. (**B**) BMOE cross-linking of strains carrying the head engagement sensor mutation G67C and ATPase blocking mutations. Cells were grown in LB for 1.5 h at 37 °C before cross-linking. In-gel fluorescence is shown on the left, and quantification of three technical replicates is shown on the right. Black lines indicate means, purple lines indicate standard deviations, and colored bars indicate 95 % credible intervals. (**C**) BMOE cross-linking of arm folding sensor strains carrying ATPase mutations. As in B.

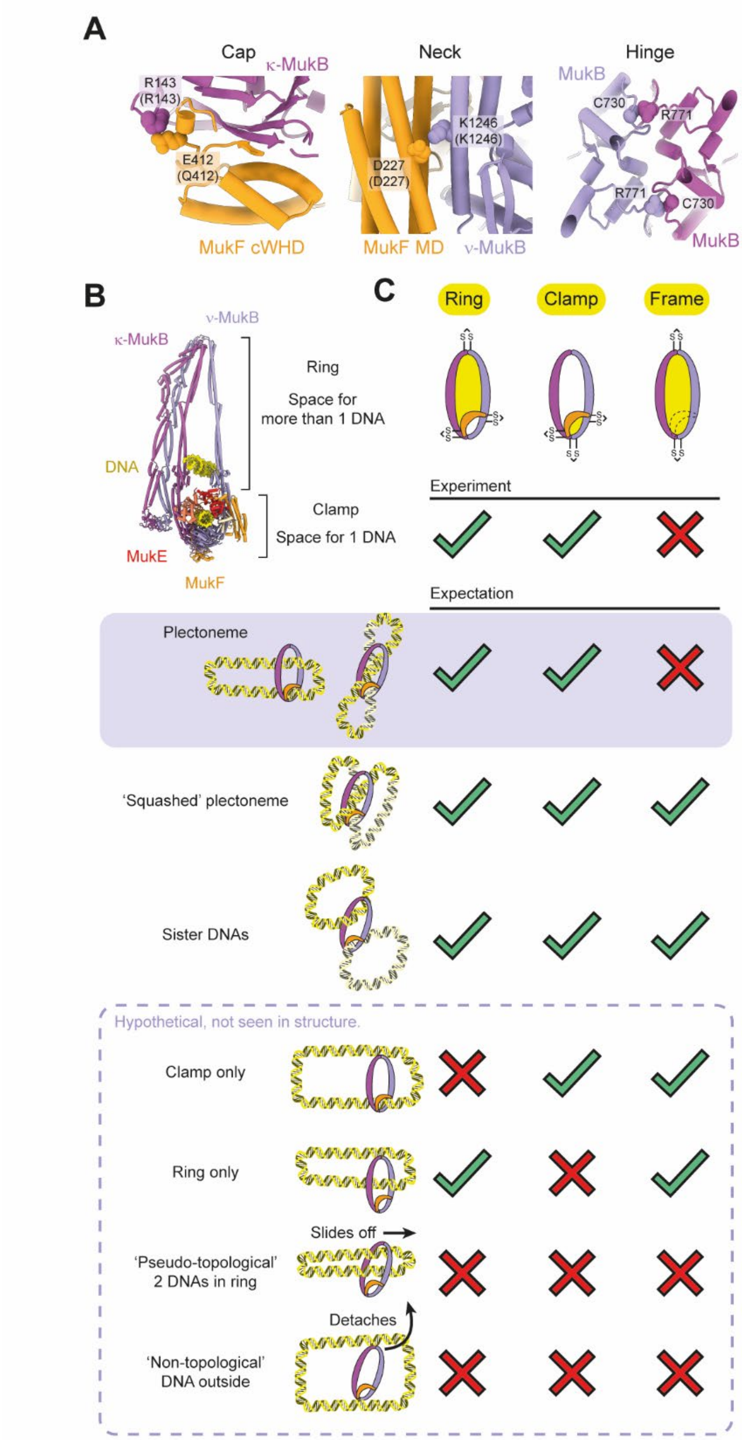

Figure S7. Chromosome entrapment by MukBEF. Related to **Figure 6**.
(**A**) Design of cap, neck and hinge cross-links. Location of residues chosen for cysteine mutagenesis at the hinge (left, PDB: 3IBP), cap (middle), and neck (right) is shown. Labels for cap and neck are shown for the *P. thracensis* structure and corresponding *E. coli* residues are in parentheses. (**B**) Space for hypothetical accommodation of additional DNA double strands. Only the ring compartment is large enough to embrace more than one DNA.
(**C**) Comparison of experimentally observed and expected catenanes for different DNA binding topologies. The topology consistent with the experimental data is highlighted. For clarity, the double-locked plectoneme topology is also shown as a simplified version without

DNA crossings. Hypothetical ‘pseudo-topological’ and ‘non-topological’ DNA loops do not produce catenanes. Their tentative formation in addition to catenated forms is therefore not excluded by the data.

Table S1. Bacterial strains. Related to STAR Methods.

Strain ID	Genotype	Figures
BL21-Gold(DE3)	F-, *lon-*, *ompT-*, *hsdS*(*rb- mb-*), *dcm+*, *tet*, *gal*, λ(DE3), *endA-*, *Hte*	
C41(DE3)	F-, *ompT*, *gal*, *dcm*, *hsdSB*(*rB- mB-*), λ(DE3)	
MG1655	F-, λ-, *rph*-1, *fnr*+	
SFB018	MG1655, Δ*mukB::neoR*	S5
SFB047	MG1655, *mukB::pheS(T251A, A294G)-hygR*, pKW20 Para *lambda-red cas9 tet tracrRNA*	
SFB053	MG1655, Δ*mukFEB::pheS(T251A, A294G)-hygR*, pKW20 Para *lambda-red cas9 tet tracrRNA*	
SFB065	DH5α, pJF146 RK24 *lux apR bsd*	
SFB114	MG1655, *mukB(A304C, N857C, C1118S)-TEV-HaloTag(C61V, C262A)::neoR*	5B, S6C
SFB115	MG1655, *mukB(G67C, C1118S)-TEV-HaloTag(C61V, C262A)::neoR*	6C, S6B
SFB116	MG1655, *mukB(C1118S)-TEV-HaloTag(C61V, C262A)::neoR*	5B, S6B, S6C
SFB117	MG1655, *mukB(A304C, C1118S)-TEV-HaloTag(C61V, C262A)::neoR*	5B
SFB118	MG1655, *mukB(N857C, C1118S)-TEV-HaloTag(C61V, C262A)::neoR*	5B
SFB119	MG1655, *mukB(R771C, C1118S)-TEV-HaloTag(C61V, C262A)::neoR*	6C
SFB120	MG1655, *mukB(G67C, C1118S, E1407Q)-TEV-HaloTag(C61V, C262A)::neoR*	S6B
SFB121	MG1655, *mukB(C730S, R771C, C1118S)-TEV-HaloTag(C61V, C262A)::neoR*	6C
SFB122	MG1655, *mukB(G67C, C1118S, D1406A)-TEV-HaloTag(C61V, C262A)::neoR*	S6B
SFB126	MG1655, *mukB(A304C, N857C, C1118S, S1366R)-TEV-HaloTag(C61V, C262A)::neoR*	S6C
SFB127	MG1655, *mukB(A304C, N857C, C1118S, D1406A)-TEV-HaloTag(C61V, C262A)::neoR*	S6C
SFB128	MG1655, *mukB(A304C, N857C, C1118S, E1407Q)-TEV-HaloTag(C61V, C262A)::neoR*	S6C
SFB167	MG1655, *mukF(D227C) mukE mukB(C1118S)-TEV-HaloTag(C61V, C262A)::neoR*	6C
SFB168	MG1655, *mukB(C1118S, K1246C)-TEV-HaloTag(C61V, C262A)::neoR*	6C
SFB169	MG1655, mukF(D227C) mukE mukB(C1118S, K1246C)-TEV-HaloTag(C61V, C262A)::neoR	6C
SFB170	MG1655, *mukF(Q412C) mukE mukB(C1118S)-TEV-HaloTag(C61V, C262A)::neoR*	6C
SFB171	MG1655, *mukF(Q412C) mukE mukB(R143C, C1118S)-TEV-HaloTag(C61V, C262A)::neoR*	6C
SFB172	MG1655, *mukF(Q412C) mukE mukB(R143C, R771C, C1118S)-TEV-HaloTag(C61V, C262A)::neoR*	6D, 6C
SFB173	MG1655, *mukF(D227C, Q412C) mukE mukB(R143C, C1118S, K1246C)-TEV-HaloTag(C61V, C262A)::neoR*	6D, 6C
SFB174	MG1655, *mukF(D227C, Q412C) mukE mukB(R143C, R771C, C1118S, K1246C)-TEV-HaloTag(C61V, C262A)::neoR*	6C-F, 6H
SFB180	MG1655, *mukF(D227C, Q412C) mukE mukB(R143C, R771C, C1118S, K1246C)::pheS(T251A, A294G)-hygR*, pKW20 Para *lambda-red cas9 tet tracrRNA*	
SFB183	MG1655, *mukB(R143C, C1118S)-TEV-HaloTag(C61V, C262A)::neoR*	6C
SFB184	MG1655, *mukF(D227C) mukE mukB(R771C, C1118S, K1246C)-TEV-HaloTag(C61V, C262A)::neoR*	6H, 6C
SFB188	MG1655, *mukB(G67C, R771C, C1118S)-TEV-HaloTag(C61V, C262A)::neoR*	6C, 6F
SFB190	MG1655, *mukF(D227C, Q412C) mukE mukB(R143C, R771C, C1118S, K1246C, D1406A)-TEV-HaloTag(C61V, C262A)::neoR*	6D, 6H
SFB191	MG1655, *mukF(D227C, Q412C) mukE mukB(R143C, R771C, C1118S, K1246C, E1407Q)-TEV-HaloTag(C61V, C262A)::neoR*	6D
SFB192	MG1655, *mukB(G67C, R771C, C1118S, D1406A)-TEV-HaloTag(C61V, C262A)::neoR*	6F
SFB193	MG1655, *mukB(G67C, R771C, C1118S, E1407Q)-TEV-HaloTag(C61V, C262A)::neoR*	6F
SFB202	MG1655, Δ*matP*, *mukF(D227C, Q412C) mukE mukB(R143C, R771C, C1118S, K1246C)-TEV-HaloTag(C61V, C262A)::neoR*	6H
SFB203	MG1655, *mukF(D227C, Q412C) mukE mukB(G67C, R143C, C1118S, K1246C)-TEV-HaloTag(C61V, C262A)::neoR*	6C, 6E
SFB204	MG1655, *mukF(D227C, Q412C) mukE mukB(G67C, R143C, C1118S, K1246C, D1406A)-TEV-HaloTag(C61V, C262A)::neoR*	6E
SFB205	MG1655, *mukF(D227C, Q412C) mukE mukB(G67C, R143C, C1118S, K1246C, E1407Q)-TEV-HaloTag(C61V, C262A)::neoR*	6E
SFB206	MG1655, *mukF(D227C, Q412C) mukE mukB(G67C, R143C, C1118S)-TEV-HaloTag(C61V, C262A)::neoR*	6C
SFB207	MG1655, *mukF(D227C, Q412C) mukE mukB(G67C, C1118S, K1246C)-TEV-HaloTag(C61V, C262A)::neoR*	6C

Table S2. Plasmids. Related to STAR Methods.

ID	Name	Description	Source
pFB017	pET-Gold1 *ccdB*	GoldenGate *BsmB*I acceptor plasmid	This study
pFB083	pGEX GST-hSENP1	T7 expression plasmid for producing GST-tagged hSENP1	Komander lab
pFB287	pET-Gold1 'mukB-TEV-HaloTag(C61V, C262A) *neoR*	Module for targeting of the *mukFEB* locus (*Bsa*I donor)	This study
pFB377	pCONEX-Gate4 CRISPR(mukFEB) *ccdB*	Shuttle plasmid for targeting of the *mukFEB* locus (*Bsa*I acceptor)	This study
pFB403	pET-Gate2 *Pth* MukF MukE His6-SUMO-MukB	T7 expression plasmid for producing SUMO-tagged MukBEF	This study
pFB411	pCONEX-Gate4 CRISPR(mukFEB cloDF13) *ccdB*	Shuttle plasmid for targeting of the *mukFEB* locus (*Bsa*I acceptor); crRNA targets pKW20 plasmid	This study
pFB448	pCONEX-Gate4 BsmBI *ccdB*	Shuttle plasmid for conjugative gene targeting; *BsmB*I acceptor site for inserting custom spacers	This study
pFB449	pCONEX-Gate5 CRISPR(mukB cloDF13) *ccdB*	Shuttle plasmid for targeting of *mukB* (*Bsa*I acceptor)	This study
pFB468	pET-Gate2 *Pth* His6-SUMO-MukB	T7 expression plasmid for producing MukB	This study
pFB469	pET-Gold1 *Pth* MatP	T7 expression plasmid for producing MatP	This study
pFB485	pET-Gate2 *Pth* His6-SUMO-MukB(E1407Q)	T7 expression plasmid for producing SUMO-tagged MukB(E1407Q)	This study
pFB486	pET-Gate2 *Pth* MukFE	T7 expression plasmid for producing MukFE	This study
pFB502	pET-Gold1 (3-723)mukB(R143C)	Module for targeting of the *mukFEB* locus (*Bsa*I donor)	This study
pFB507	pET-Gold1 mukF(D227C, Q412C)EB'	Module for targeting of the *mukFEB* locus (*Bsa*I donor)	This study
pFB508	pET-Gold1 (723-1480)mukB(R771C, C1118S, K1246C)	Module for targeting of the *mukFEB* locus (*Bsa*I donor)	This study
pJF146	RK24 *lux apR bsd*	RK2 conjugation machinery; NCBI: MK809154.1	Fredens et al., 2019
pKW20	Para *lambda-red cas9 tet tracrRNA*	REXER helper plasmid; NCBI: MN927219.1	Wang et al., 2016
