# Supplementary material for: Cryo-EM structure of MukBEF reveals DNA loop entrapment at chromosomal unloading sites
Source: Mol Cell. 2021 Dec 2;81(23):4891–4906.e8. doi: 10.1016/j.molcel.2021.10.011 (PMC8669397; doi:10.1016/j.molcel.2021.10.011)
Supplement: Document S2. Article plus supplemental information [file mmc6.pdf]

Article

Molecular Cell

Cryo-EM structure of MukBEF reveals DNA loop entrapment at chromosomal unloading sites

Graphical abstract

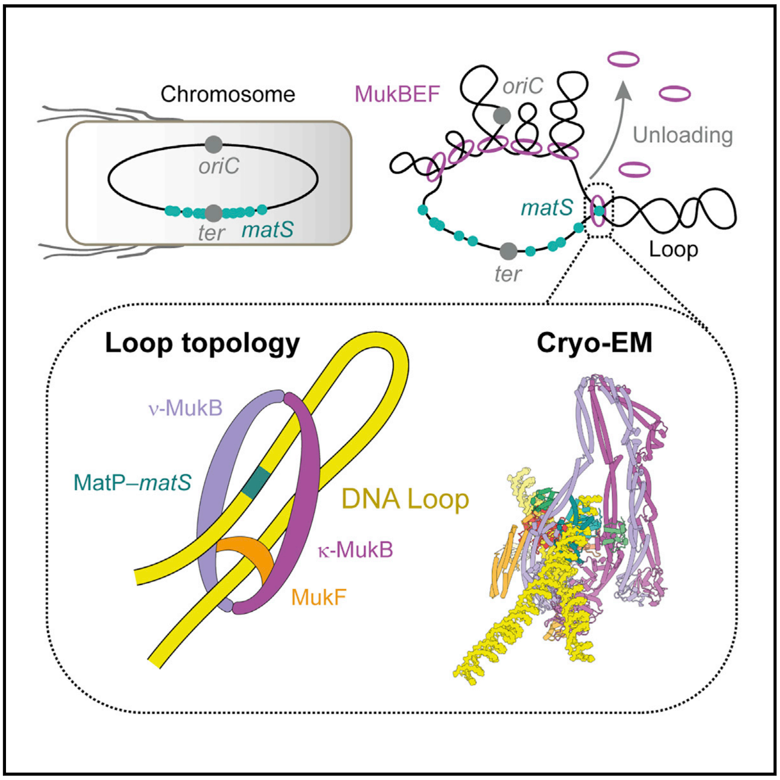

Authors

Frank Bürmann, Louise F.H. Funke, Jason W. Chin, Jan Löwe

Correspondence

fburmann@mrc-lmb.cam.ac.uk (F.B.), jyl@mrc-lmb.cam.ac.uk (J.L.)

In brief

The SMC complex MukBEF organizes bacterial chromosomes into large DNA loops. In this article, Bürmann et al. report the cryo-EM structure of MukBEF bound to its unloading factor, MatP, and two DNA segments corresponding to the arms of a loop. The article provides insights into how SMC complexes topologically entrap DNA and unload from chromosomes.

Highlights

- Complete atomic structures of the bacterial SMC complex MukBEF on and off DNA
- MukBEF entraps two DNA double helices when bound to the unloader MatP
- *In vivo* topology of DNA loop entrapment determined by cysteine cross-linking
- Arms of the DNA loop thread through separate compartments of MukBEF

Bürmann et al., 2021, Molecular Cell *81*, 4891–4906
December 2, 2021 © 2021 MRC Laboratory of Molecular Biology.
Published by Elsevier Inc.
https://doi.org/10.1016/j.molcel.2021.10.011

Check for updates

Molecular Cell

CellPress
OPEN ACCESS

Article

Cryo-EM structure of MukBEF reveals DNA loop entrapment at chromosomal unloading sites

Frank Bürmann,[1,*] Louise F.H. Funke,[2] Jason W. Chin,[2] and Jan Löwe[1,3,*]
[1]MRC Laboratory of Molecular Biology, Structural Studies Division, Cambridge Biomedical Campus, Cambridge, UK
[2]MRC Laboratory of Molecular Biology, Protein and Nucleic Acid Chemistry Division, Cambridge Biomedical Campus, Cambridge, UK
[3]Lead contact
*Correspondence: fburmann@mrc-lmb.cam.ac.uk (F.B.), jyl@mrc-lmb.cam.ac.uk (J.L.)
https://doi.org/10.1016/j.molcel.2021.10.011

SUMMARY

The ring-like structural maintenance of chromosomes (SMC) complex MukBEF folds the genome of *Escherichia coli* and related bacteria into large loops, presumably by active DNA loop extrusion. MukBEF activity within the replication terminus macrodomain is suppressed by the sequence-specific unloader MatP. Here, we present the complete atomic structure of MukBEF in complex with MatP and DNA as determined by electron cryomicroscopy (cryo-EM). The complex binds two distinct DNA double helices corresponding to the arms of a plectonemic loop. MatP-bound DNA threads through the MukBEF ring, while the second DNA is clamped by the kleisin MukF, MukE, and the MukB ATPase heads. Combinatorial cysteine cross-linking confirms this topology of DNA loop entrapment *in vivo*. Our findings illuminate how a class of near-ubiquitous DNA organizers with important roles in genome maintenance interacts with the bacterial chromosome.

INTRODUCTION

Associations between molecules due to their topology are known as mechanical bonds (Stoddart, 2009). In eukaryotes as well as prokaryotes, ring-like structural maintenance of chromosomes (SMC) complexes are thought to structure chromosomes via mechanical bonds with DNA (also referred to as "DNA entrapment") and active DNA loop extrusion (Davidson and Peters, 2021; Hassler et al., 2018; Mäkelä and Sherratt, 2020a; Nasmyth, 2001; Yatskevich et al., 2019). These activities have been suggested to enable or facilitate processes such as lengthwise condensation of chromosomes, sister chromatid cohesion, regulation of interactions between enhancers and distant promoters, disentangling of replicated DNA by topoisomerases, DNA recombination, and DNA double-strand break repair. Support for DNA entrapment, whereby the SMC complex encircles the nucleic acid polymer, comes from experiments probing DNA association after high salt treatment or, more stringently, chemical circularization and denaturation of the complex (Cuylen et al., 2011; Haering et al., 2008; Ivanov and Nasmyth, 2005; Kanno et al., 2015; Murayama and Uhlmann, 2014; Niki and Yano, 2016; Wilhelm et al., 2015). How DNA entrapment is achieved on the molecular level is less understood, as SMC complexes contain multiple topological compartments that can or could accommodate one or more DNA double strands (Chapard et al., 2019; Collier et al., 2020; Higashi et al., 2020; Shi et al., 2020; Vazquez Nunez et al., 2019).

At the core of SMC complexes—such as cohesin, condensin, Smc5-6, Smc-ScpAB, MksBEF, and MukBEF—is a tripartite ring composed of two SMC proteins and a kleisin. SMC proteins contain a 50-nm-long intra-molecular anti-parallel coiled-coil "arm," which can fold over at an "elbow" in MukBEF, cohesin, and condensin (Bürmann et al., 2019; Lee et al., 2020; Niki et al., 1992). The arm separates a "hinge" dimerization domain from an ABC-type ATPase "head" domain, which undergoes cycles of ATP-dependent dimerization (called "engagement"), ATP hydrolysis, and disengagement (Hopfner et al., 2000). The kleisin bridges hinge-dimerized SMC proteins in an asymmetric arrangement, whereby its N- and C-terminal domain bind the SMC "neck" and "cap" surfaces, respectively (Bürmann et al., 2013; Gligoris et al., 2014; Haering and Gruber, 2016; Haering et al., 2004; Schleiffer et al., 2003; Woo et al., 2009; Zawadzka et al., 2018). The neck is located at the very head-proximal region of the arm, whereas the cap is part of the head domain. The neck-bound SMC is designated ν-SMC (nu for neck), and the cap-bound subunit is designated κ-SMC (kappa for cap).

MukBEF is the SMC complex of *E. coli* and other enterobacteria. Its SMC subunit is MukB, which associates with the kleisin MukF (Woo et al., 2009; Zawadzka et al., 2018). MukF binds the dimeric KITE protein MukE, which is structurally related to ScpB of prokaryotic Smc-ScpAB and Nse1-3 of the Smc5-6 complex (Palecek and Gruber, 2015). $MukB_2E_2F$ assemblies (hereafter "MukBEF monomers") dimerize via MukF (Fennell-Fezzie et al., 2005; Woo et al., 2009) into $MukB_4E_4F_2$ complexes

© 2021 MRC Laboratory of Molecular Biology. Published by Elsevier Inc.
This is an open access article under the CC BY license (http://creativecommons.org/licenses/by/4.0/).

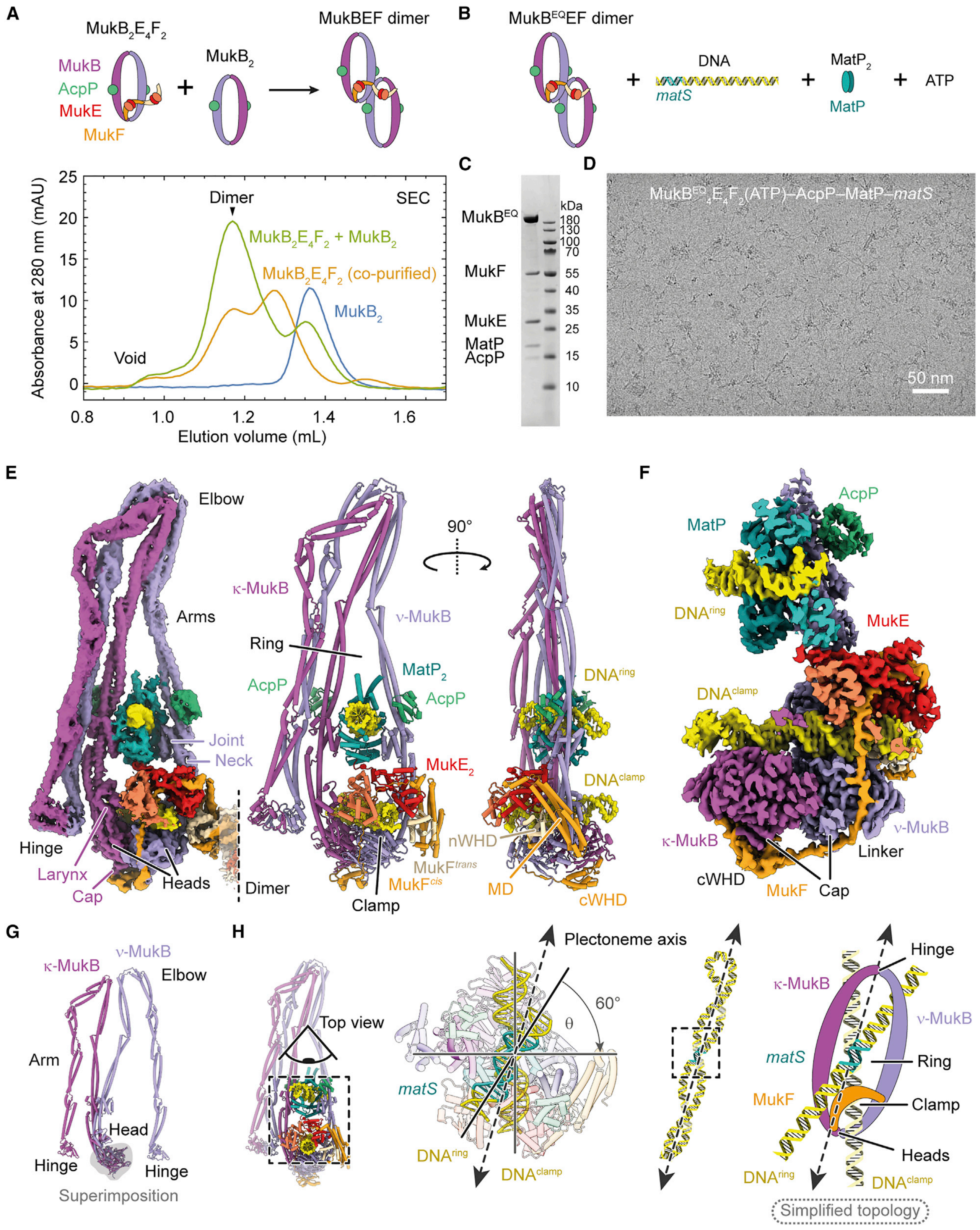

(legend on next page)

CellPress
OPEN ACCESS

(also called "MukB dimers of dimers"; hereafter simply "MukBEF dimers"), which are the functional forms (Badrinarayanan et al., 2012; Rajasekar et al., 2019).

MukBEF organizes large fractions of the *E. coli* chromosome and is essential for chromosome segregation and cell survival under conditions of fast growth (Danilova et al., 2007; Hiraga et al., 1989; Lioy et al., 2018). In the replication terminus macrodomain (*Ter*), MukBEF activity is suppressed by unloading at *matS* sites, which are the signature sequences of *Ter* (Lioy et al., 2018; Mäkelä and Sherratt, 2020b; Mercier et al., 2008; Nolivos et al., 2016). Dedicated removal of SMC complexes from the replication terminus appears widespread among bacteria, as *B. subtilis* Smc-ScpAB is unloaded by the chromosome resolvase XerD (Karaboja et al., 2021). Unloading of MukBEF drastically changes the loop-size distribution of *Ter* compared to other chromosomal macrodomains, a process that depends on the *matS*-binding protein MatP (Lioy et al., 2018). Although MukBEF function relies on the full ATPase cycle (Badrinarayanan et al., 2012; Woo et al., 2009), association with *matS* requires ATP-dependent head engagement only (Nolivos et al., 2016). Head engagement and ATP hydrolysis have been suggested to mediate unloading of cohesin in eukaryotes, which involves dissociation of the neck/kleisin interface (Chan et al., 2012; Muir et al., 2020; Murayama and Uhlmann, 2015). This interface also disengages during the ATPase cycle of condensin, raising the possibility that SMC complexes may use related mechanisms for DNA unloading (Hassler et al., 2019).

Here, using electron cryomicroscopy (cryo-EM) single-particle analysis, we discovered that MukBEF entraps two distinct DNA double helices when bound to the unloader MatP. The DNAs reside in separate compartments, which are located inside the large circumference of the tripartite ring and in a much smaller clamp at the ATPase heads. Topological mapping by chemical circularization of endogenous MukBEF in cells suggests that these compartments enclose each arm of a DNA loop *in vivo*. Our findings illuminate how MukBEF can entrap DNA loops and how these loops are primed for unloading from the complex.

RESULTS

MukBEF-MatP entraps two DNA double helices in topologically separate compartments

Initial attempts to determine the structure of *E. coli* MukBEF were unsuccessful. We then recombinantly produced MukBEF complexes from 11 different species in *E. coli* and identified the complex from *Photorhabdus thracensis* as a suitable candidate for structure determination by cryo-EM. The complex has 78% sequence identity to its *E. coli* homolog and stably co-purified with the *E. coli* acyl-carrier protein AcpP, an essential protein that is a binding partner of *E. coli* MukBEF (Niki et al., 1992; Prince et al., 2021). *E. coli* AcpP is 85% identical to *P. thracensis* AcpP. The purified complex had an estimated stoichiometry of $MukB_2E_4F_2$-$AcpP_2$, which is a $MukB_2$-$AcpP_2$ unit short of the $MukB_4E_4F_2$ dimer expected from quantitative live-cell fluorescence microscopy (Badrinarayanan et al., 2012). We reasoned that this was due to either dissociation of MukBEF dimers during purification or overproduction of MukEF, leading to saturation and incomplete assembly of the complex. We therefore titrated the preparation with $MukB_2$-$AcpP_2$ to reconstitute intact $MukB_4E_4F_2$ dimers. This almost quantitatively shifted MukBEF to a smaller elution volume in size-exclusion chromatography (SEC), indicating the formation of the physiological complex (Figure 1A).

To gain insights into how MukBEF interacts with *matS* sites during chromosomal unloading, we purified *P. thracensis* MatP, identified a cognate high-affinity *matS* site (Figure S1), and introduced the E1407Q mutation (hereafter, $MukB^{EQ}$) into MukB to slow down ATP hydrolysis (Woo et al., 2009). We then reconstituted a complex of $MukB^{EQ}$EF dimers, MatP, and an 80-bp DNA oligonucleotide containing *matS* close to one end in the presence of ATP and magnesium ions (Figures 1B and 1C). The sample was then imaged by cryo-EM in vitreous ice (Figure 1D).

We obtained a reconstruction of the DNA-bound MukBEF monomer part at an overall nominal resolution of 4.6 Å (Figures 1E and S2). Focused classification and refinement produced a map of 3.1 Å resolution for the more rigid head module, with clearly resolved ATP and magnesium ions mediating head engagement (Figures 1F and S3A). This allowed the construction of a complete atomic model for the complex, facilitated by previous crystallographic information for individual parts (PDB: 3EUH, 3EUJ, 3IBP, 3VEA, 6DFL, 6H2X) (Bürmann et al., 2019; Dupaigne et al., 2012; Kreamer et al., 2018; Li et al., 2010; Woo et al., 2009).

MukBEF adopts a compact and highly asymmetric conformation, with its ATPase heads bridged by the kleisin MukF (Figure 1E; Video S1). The heads and hinge of MukB are brought into proximity by folding at the elbow. In addition to the elbow and the "joint" at the heads (Diebold-Durand et al., 2017), the MukB arms contain several other coiled-coil discontinuities

Figure 1. Cryo-EM structure of MukBEF-MatP bound to two distinct DNA double helices
(A) Reconstitution of MukBEF dimers. Co-purified MukBEF and free MukB were mixed (top) and resolved by SEC (bottom).
(B) Composition of the MukBEF-MatP-*matS* sample used for structure determination.
(C) Coomassie stained SDS-PAGE gel of the reconstituted complex used for cryo-EM.
(D) Example micrograph of the sample used for structure determination.
(E) A 4.6-Å-resolution cryo-EM density map (left, EMDB: EMD-12657) and complete atomic model (middle, right, PDB: 7NYX) of the DNA-bound MukBEF-MatP monomer.
(F) Slice through a 3.1-Å-resolution cryo-EM density map of the DNA-binding region of MukBEF-MatP (EMDB: EMD-12656, PDB: 7NYW).
(G) κ-MukB and ν-MukB superimposed on the head domain. The arms adopt radically different conformations.
(H) DNA binding topology on plectonemic loops inferred from the DNA crossing angle Θ. The crossing angle convention employed by Rawdon et al. (2016) is used. The schematic on the right shows the simplified topology used for clarity throughout, with the in-reality elbow-folded conformation flattened into a ring.
See also Figures S1–S3 and Video S1.

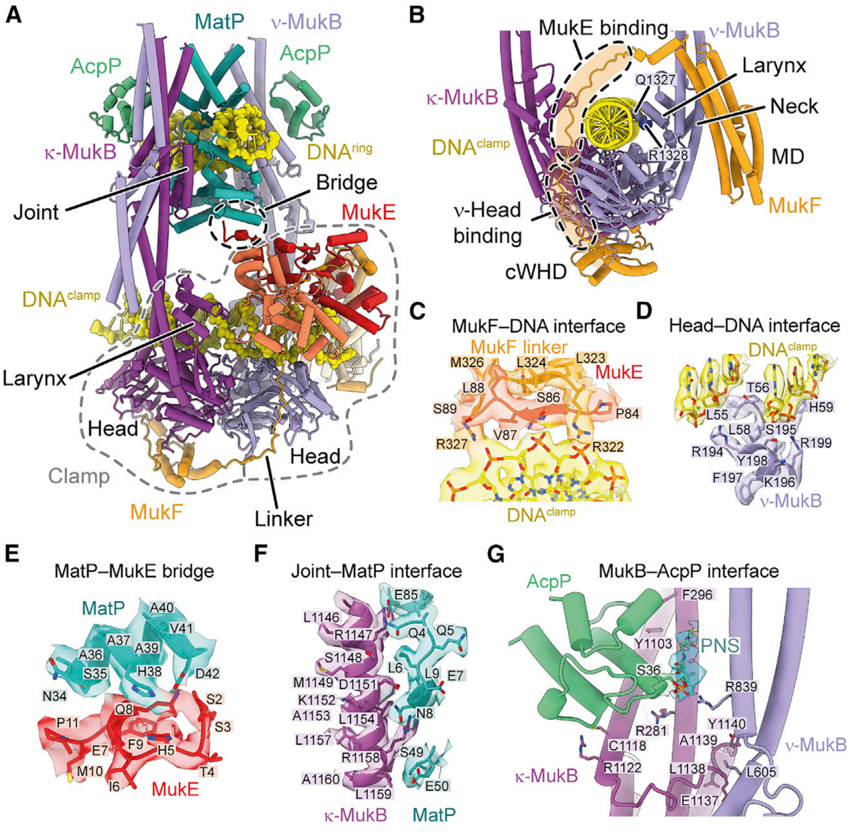

Figure 2. DNA binding and subunit interfaces of the MukBEF-MatP complex

(A) Model of the DNA-bound head module (PDB: 7NYW).

(B) Path of the kleisin MukF and DNA contacts of the MukB larynx.

(C) Interface between the MukF linker and the clamped DNA.

(D) Interface between the top surface of the ν-MukB head and the clamped DNA.

(E) Interface between MukE and MatP.

(F) Interface between the MukB joint and MatP.

(G) Interfaces between MukB and AcpP and between the κ-MukB joint and the hinge-proximal arm of ν-MukB.

See also Figures S2 and S3 and Video S1.

(Weitzel et al., 2011). Plasticity in these regions allows κ-MukB and ν-MukB to adopt radically different conformations and, thus, break homodimer symmetry (Figure 1G).

The head-proximal arms of MukB are open to allow accommodation of two distinct DNA double helices (Figures 1E, 1F, 1H, and 2A). One DNA is bound by MatP and threads through the inter-arm space near the joint. The other is clamped by the MukB heads, MukF, and MukE. The kleisin MukF is resolved between residues 10 and 440, which represent 98% of the protein (Figures 1F and 2B). It partitions the two DNAs into topologically separate compartments: the "ring" delimited by MukF and the MukB arms and the "clamp" delimited by MukF and the MukB heads (Figures 1E and 1H). The DNA double helices have a crossing angle of 60°, which is close to what has been estimated for negatively supercoiled plectonemes (Rawdon et al., 2016). This suggests that in the context of an intact chromosome, they may originate from a single plectonemic loop, with MukBEF binding across the long axis of the loop (Figure 1H). This hypothesis will be explored below.

The clamp contacts DNA across all core subunits

The clamp has a highly asymmetric architecture imposed by the kleisin MukF. MukF comprises a C-terminal winged-helix domain (cWHD), a four-helix bundle forming the middle domain (MD), and an N-terminal WHD (nWHD). The cWHD and MD are connected by a 64-amino-acid linker, which contains the MukE binding sites (Figure 2B). The cWHD binds the cap of its cognate κ-SMC (Figure 1F), similar to the corresponding interface in other SMC complexes (Bürmann et al., 2013; Haering et al., 2004; Hassler et al., 2019; Woo et al., 2009). The MD, which can bind the MukB neck (Zawadzka et al., 2018), is in a position roughly equivalent to binding sites between the kleisins and ν-SMCs of cohesin, condensin, and Smc-ScpAB (Bürmann et al., 2013; Gligoris et al., 2014; Hassler et al., 2019) (Figure S3B). The MD is, however, structurally unrelated to the N-terminal α-helical domain (nHD) of these kleisins.

MukB forms a homodimer; thus, both MukB subunits contain MukF binding sites at cap and neck. However, the ν-MukB does not form the cap/cWHD interface, and κ-MukB does not associate with an MD. This asymmetric configuration is enabled by two separate steric occlusion mechanisms. At the ν-MukB cap, binding of the MukF linker to ν-MukB prevents binding of a cWHD, as has been observed before (Woo et al., 2009) (Figures 1F and 2B). In addition, the neck of κ-MukB is occluded by the hinge (Figure 1E), which prevents binding of an MD. These mechanisms preclude recruitment of additional MukEF subunits to the complex and, thus, prevent chaining of MukBEF monomers into higher-order polymers.

Within the clamp, all core subunits of MukBEF are in contact with DNA. The MukF linker is guided over the clamped DNA by the MukE dimer, which also binds DNA along its central cleft (Figures 2A and 2B). The linker itself contacts the phosphate backbone with R322 and R327 (Figure 2C). The MukB heads bind the clamped DNA along their top surface (Figure 2D). The "larynx" of ν-MukB provides additional DNA contacts with Q1327 and R1328 (Figure 2B). This globular domain is situated at the base of the neck and is not present in most other SMC proteins (Figure S3C). Interestingly, the nHD of cohesin's kleisin Rad21 provides DNA contacts that are located in a position similar to the larynx (Figure S3B).

The overall architecture of the MukBEF clamp appears analogous to what has been observed for the nuclease clamp in SbcCD (Rad50-Mre11) and the HAWK clamp in cohesin (Collier et al., 2020; Higashi et al., 2020; Kӓshammer et al., 2019; Shi et al., 2020) (Figure S3D). The KITE MukE is unrelated to any

CellPress
OPEN ACCESS

subunit in these complexes; however, other KITE-based SMC complexes, such as Smc5-6 and Smc-ScpAB, may clamp DNA in a manner similar to MukBEF. We conclude that DNA binding on top of the ATPase heads is a common principle between different SMC complexes, whereas the non-SMC subunits create structurally divergent but topologically equivalent clamps.

MatP and MukE bridge the two DNAs

Whereas the clamped DNA is contacted by all core subunits of the complex, the DNA inside the ring is mostly bound by MatP, with only K1178 in the MukB joint contacting the phosphate backbone. The MatP dimer recognizes *matS* by inserting its $\alpha 4$ and $\beta 1$ elements into the major grove, as has been determined for MatP-*matS* complexes in isolation (Dupaigne et al., 2012) (Figure S1C). The C-terminal tetramerization tail of MatP, however, is not visible and is likely disordered, consistent with the finding that it is not required for MukBEF-related functions (Nolivos et al., 2016).

Interestingly, one of the MatP monomers forms a contact with one of the MukE monomers (Figures 2A and 2E). The DNAs in the ring and clamp are, thus, physically linked via MukE and MatP. The bridge is formed by residues between H38 and D42 in MatP and an N-terminal tail of MukE (Figure 2E). The latter involves residues between S2 and Q8, which are disordered in the second MukE subunit and in previous crystal structures (Gloyd et al., 2011; Woo et al., 2009). The bridge interface is small and likely prone to dissociation, consistent with the finding that recombinantly overexpressed MukEF does not co-immunoprecipitate with purified MatP (Nolivos et al., 2016). This suggests that the bridge may have a transient role during unloading and dissociates once the reaction is complete, permitting the release of MukBEF from *matS* sites.

The MukB joint is an interaction hub for MatP, AcpP, and the hinge-proximal MukB arm

The joint of MukB is located at a central region of the complex. It is formed by an 84-amino-acid insertion into the C-terminal coiled-coil strand and forms a slightly larger domain than the joints found in other SMC proteins (Figure S3C). The joint binds and positions MatP between the MukB arms (Figures 2A and 2F). This interface is much larger than the MukE-MatP bridge and likely provides the major binding energy for association with MatP. The joint also provides a docking site for the hinge-proximal arm, with an interface formed between residues 602–609 of the ν-MukB arm and residues 1136 –1140 of the κ-MukB joint (Figure 2G). This likely contributes to stabilization of the elbow-folded conformation of MukBEF.

AcpP binds MukB close to the joint between R281 and F296 on the N-terminal coiled-coil helix and Y1103 and R1122 on the C-terminal helix (Figure 2G). Weak density protrudes from S36 of AcpP, which we have modeled as phosphopantetheine (PNS), the prosthetic group of AcpP that is covalently bound to S36 and can flip out its core upon association with binding partners (Cronan, 2014). At the κ-MukB binding site, PNS projects toward the space between the head-proximal arm of κ-MukB and the hinge-proximal arm of ν-MukB. The phosphate group of PNS is in contact with R839 of ν-MukB. PNS is modified with acyl moieties during fatty acid synthesis, and although the biological function of AcpP within the MukBEF complex is unclear, it may have a regulatory role coupling metabolism to chromosome organization (Gully et al., 2003). Because of its position near the joint-arm contact, it is possible that AcpP, perhaps controlled by its modification state, could have an influence on the elbow-folded state of MukBEF.

The joint is situated near the heads and is, thus, expected to be a central conduit for conformational changes imposed by the ATPase cycle. Consistent with this idea, AcpP binding at the joint strongly increases MukBEF ATPase activity (Prince et al., 2021). Release of MukBEF from *matS* will likely require detachment from MatP; hence, MatP binding at the joint seems ideal for regulation by the ATPase cycle, as will be explored below.

Architecture of apo-MukBEF and the MukBEF dimer

In the same sample that produced reconstructions of MukBEF bound to MatP-*matS*, we also observed particles with disengaged heads and that were neither DNA nor MatP bound (Figure 3A). Although the map was resolved to only 6.8 Å, which prevented determination of the nucleotide state, it was very similar to exploratory reconstructions of nucleotide-free MukBEF (Figure S2A). Hence, we refer to it as the “apo state.” The apo complex is comparable in size and shape to apo yeast condensin, with arms fully juxtaposed (Figure S3E). The apo clamp is more flexible because it is not held in place by ATP and DNA, but clear density was observed at lower contour levels that allowed unambiguous positioning of MukEF. The map also revealed density for the second monomer within the context of a MukBEF dimer. Further classification produced a low-resolution map for the apo dimer, from which we obtained a model by rigid body fitting (Figure 3B; Video S2).

The MukBEF dimer is held together by an extensive interface between MukF’s MD and nWHD, which was observed previously by crystallography (Fennell-Fezzie et al., 2005; Woo et al., 2009). The two MukBEF monomers associate head to head with their MukEF subunits on the same face of the dimer. As dictated by its symmetry, the complex thus has a front and back (Figure 3B).

In addition to dimers in the apo state, dimers associated with MatP and DNA were readily resolved (Figure 3C; Video S2). Because we positioned the *matS* site close to one end of the 80-bp DNA used for sample preparation, two dimers were able to associate with four DNAs in a “tetrad” arrangement. Different classes of tetrads allowed us to assess the distance between the two dimers. We observed dimers distantly bridged by the DNA molecules (Figure 3C) and closely apposed (Figure 3D). This is explained by sequence-independent binding of the clamp, which can associate with any position along the DNA double strand (Figure 3E). Assuming that the clamp binds DNA not only during unloading at *matS*, but also during a tentative translocation reaction, it may step or slide along the DNA track. Since the DNAs are roughly aligned with the dimer symmetry axis, it is conceivable that DNA translocation may operate along this axis.

Conformational changes associated with unloading

The MukB^{EQ}EF-MatP-*matS* complex is prevented from hydrolysing ATP and shows a state prior to unloading. The apo form, however, lacks both MatP and DNA and therefore represents

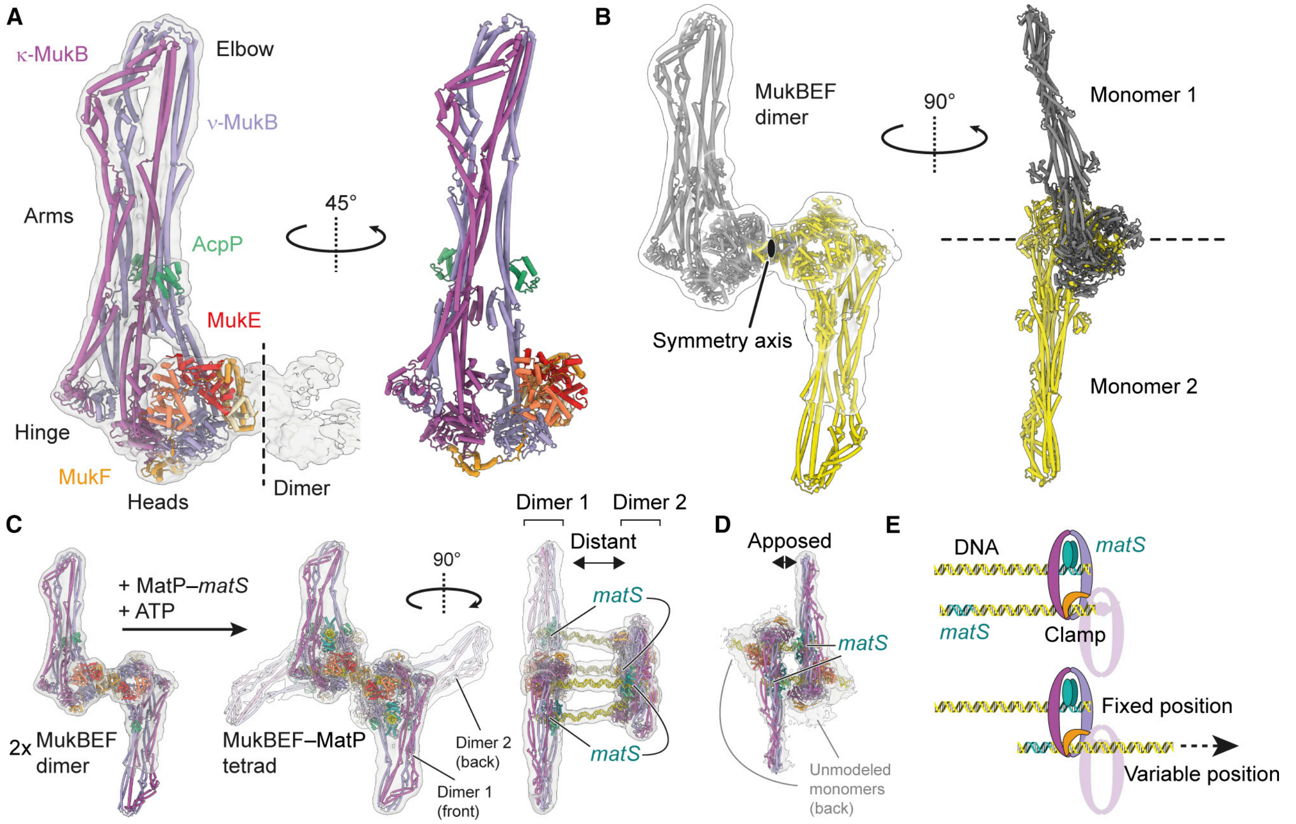

Figure 3. Architecture of apo-MukBEF and the MukBEF dimer

(A) Model of the apo-MukBEF monomer (PDB: 7NYY) and 6.8-Å cryo-EM density (EMDB: EMD-12658) at low contour level.

(B) Model for the apo-MukBEF dimer (PDB: 7NZ4) and 13-Å cryo-EM density (EMDB: EMD-12664).

(C) The 11-Å cryo-EM density (EMDB: EMD-12662) and model (PDB: 7NZ2) for two MukBEF dimers bridged by four MatP-DNA complexes (“MukBEF tetrad”). The apo-MukBEF dimer is shown on the left.

(D) The 11-Å cryo-EM density (EMDB: EMD-12663) and model (PDB: 7NZ3) for a MukBEF tetrad with closely apposed dimers. Only one monomer for each MukBEF dimer was modeled due to weak density for their partner monomers.

(E) Schematic for variable positioning of the clamp DNA binding site, as shown in (C) and (D). Only a single MukBEF dimer and only two of the four DNAs are shown for clarity.

See also Figures S2 and S3 and Video S2.

the result of a completed unloading reaction. Comparison of the two states should yield insights into conformational changes that take place during MatP-dependent DNA exit.

Sub-classification of the cryo-EM dataset revealed additional forms of MukBEF-MatP with arms in different states of openness (Figure 4A). This suggests that the arms can gradually “zip up,” similar to what has been proposed for Smc-ScpAB based on distance measurements by electron paramagnetic resonance (Vazquez Nunez et al., 2021). The most open class has arms unzipped up to the elbow, and the most closed one is the apo state. In the apo state, the heads disengage and tilt, and the joints and larynx become closely juxtaposed (Figure 4B; Video S3). This occludes the binding sites for both MatP-*matS* and the clamped DNA and strongly supports the idea that ATP hydrolysis promotes dissociation from MatP-*matS* and DNA unloading.

Conformational changes associated with the release of nucleotide and DNA propagate through the whole complex and can be observed even in the hinge-proximal coiled-coil (Figure 4C). The hinge follows the tilting κ-MukB neck during DNA unloading, and the hinge-proximal arm changes from a straightened conformation to a strongly curved one. This indicates that the arm stores parts of the binding energy provided by ATP, MatP, and DNA as elastic energy, similar to a spring. This energy may be harnessed to expel MatP and DNA after ATP hydrolysis. Although the apo state was readily resolved, we did not observe classes with only one DNA bound in either compartment, nor with only MatP bound at the joints, nor with heads engaged but no DNA bound. This suggests that the binding sites cooperate and are regulated by ATP hydrolysis.

In cohesin, DNA unloading proceeds via opening of the interface between the kleisin Scc1 and the ν-SMC Smc3 (Chan et al., 2012; Muir et al., 2020; Murayama and Uhlmann, 2015). In MukBEF, the corresponding interface, which we name “neck gate,” is formed by the neck of ν-MukB and the MD of one MukF (MukF^{cis}) together with the nWHD of the second MukF (MukF^{trans}) (Figure 4D). Intriguingly, the neck gate is open by a

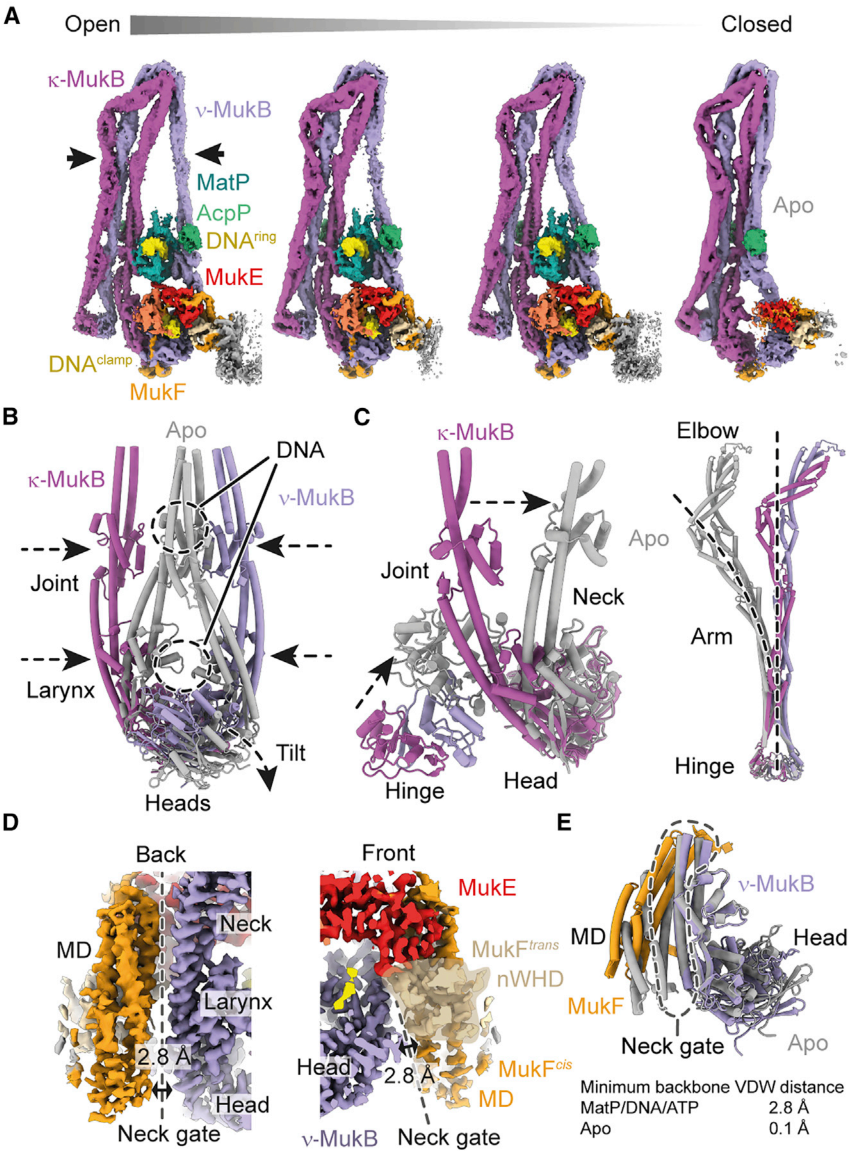

Figure 4. Conformational changes associated with release of MatP/DNA/ATP

(A) Cryo-EM densities for the MukBEF-MatP-DNA complexes with different arm conformations (EMDB: EMD-12660, EMD-12659, EMD-12657, and EMD-12658; PDB: 7NZ0, 7NYZ, 7NYX, and 7NYY).

(B) Blocking of MatP and DNA binding sites at the MukB joint and larynx. Structures were superimposed on the ATPase domains. MatP/DNA/ATP-bound conformation is shown in color, and apo conformation is in gray.

(C) Conformational change at the MukB neck/hinge interface (left) and at the hinge-proximal arm (right). Structures were superimposed on the ATPase domain (left) or the hinge (right).

(D) Cryo-EM density at the neck gate in the MatP/DNA/ATP-bound state. The solvent accessible cleft between MukB and MukF is indicated by a double arrow.

(E) Superimposition of the neck gate in apo and MatP/DNA/ATP-bound states (top). Minimum backbone VDW distances of the interface are given (bottom).

See also Figures S2 and S3 and Video S3.

narrow cleft along the interface in the MatP/DNA-bound structure but is closed in the apo structure (Figures 4D and 4E). The minimum backbone Van-der-Waals (VDW) distance is 2.8 Å across the interface in the MatP/DNA-bound state, which is reduced to 0.1-Å minimum backbone VDW distance in the apo structure. Although the cleft is too narrow for DNA to pass through, it may represent a step toward full opening of the neck gate.

MukBEF adopts a folded conformation *in vivo*

MukBEF adopts an elbow-folded conformation, at least in its apo state and when bound to ATP, MatP, and DNA. However, the elbow has also been crystallized in an extended conformation, suggesting that MukBEF may convert to extended rods or fully open rings with disengaged arms (Bürmann et al., 2019) (Figure 5A). The hinge-proximal arms are in a closed-rod conformation in our structures, similar to those of other SMC complexes (Figure S3F), but a conformation compatible with open rings has been observed by crystallography (Li et al., 2010) (Figure S3G). Additional support for the existence of open rings comes from rotary shadowing electron microscopy experiments (Matoba et al., 2005). A closed-rod-to-open-ring transition has been proposed to drive a peristalsis-like translocation mechanism of SMC complexes (Marko et al., 2019; Minnen et al., 2016; Nomidis et al., 2021). We thus decided to clarify whether the elbow-folded conformation is abundant *in vivo* and whether it may be controlled by the ATPase cycle of MukBEF. To accomplish this, we probed the conformation of endogenous *E. coli* MukBEF by site-specific cysteine cross-linking.

To introduce multiple point mutations spread across the 8-kb chromosomal *mukFEB* locus, we used a derivative of REXER (Wang et al., 2016) (replicon excision for enhanced genome engineering through programmed recombination) (Figure S4). In addition to introducing mutations A304C and D857C into MukB to probe the folded conformation (Figure 5A), we introduced C1118S, which ablated weak background cross-linking with a small protein, possibly AcpP (Figures 2G and S5). Next, we treated the *E. coli* cells with bismaleimidoethane (BMOE), which rapidly *in vivo* cross-links closely spaced thiols such as cysteine sidechains. We then detected reaction products labeled with HaloTag-tetramethylrhodamine (TMR) by SDS-PAGE and in-gel fluorescence (Figure 5B). Residues A304C and D857C cross-linked specifically with about 40% efficiency, demonstrating that the folded conformation exists *in vivo*. The reaction efficiency was comparable to that of constitutive interfaces (see below), indicating that the folded state is abundant.

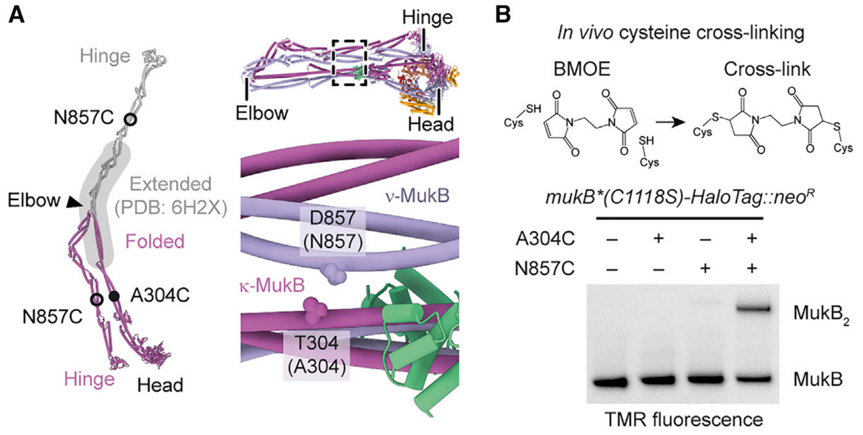

Figure 5. Detection of arm folding *in vivo*

(A) Residues employed as sensors for the folded conformation. The folded conformation and a tentative extended conformation based on the structure of the extended elbow (PDB: 6H2X) are shown on the left. A close-up on the *P. thracensis* structure is shown on the right. Corresponding *E. coli* residues are in parentheses.

(B) BMOE reaction scheme (top) and BMOE mediated *in vivo* cysteine cross-linking of *E. coli* strains carrying sensor cysteine mutations (bottom). Reaction products were detected by SDS-PAGE and in-gel fluorescence using a TMR fluorophore bound to MukB-HaloTag.

See also Figures S4–S6.

Next, we generated ATPase mutant strains S1366R (MukB^{SR}, blocking head engagement), D1406A (MukB^{DA}, blocking ATP binding), or E1407Q (MukB^{EQ}, blocking ATP hydrolysis) (Woo et al., 2009) (Figure S6A). As expected, all mutations conferred a *mukB*-null phenotype, characterized by an inability to grow on rich media at 37°C. To probe the effect of the mutations on the ATPase cycle, we then introduced G67C, which is located at the top of the MukB head and close to its symmetry mate in the second MukB (Figure S6A). This residue pair changes distance upon head engagement and should respond to alterations in the ATPase cycle. The residue cross-linked with similar efficiencies in wild-type (WT) and the ATP binding mutant MukB^{DA}, with 22% ± 1% and 21% ± 2%, respectively. The cross-linked fraction was increased to 30% ± 1% in MukB^{EQ}, indicating enhanced head engagement in this mutant (Figure S6B). As MukB^{DA} is deficient in head engagement and, thus, is an estimator for baseline cross-linking in the apo state, this suggests that a large fraction of WT MukBEF heads are disengaged. These findings are similar to what has been observed for *B. subtilis* Smc-ScpAB (Minnen et al., 2016) and confirm that the assay is able to detect conformational changes *in vivo*.

We then probed for elbow folding in MukB^{SR}, MukB^{DA}, and MukB^{EQ} strains, which resulted in similar reaction efficiencies to WT (Figure S6C). These findings suggest that the elbow-folded state of MukBEF is not controlled by steps preceding ATP hydrolysis, consistent with our structural data. However, folding could still be affected during formation of an ATP hydrolysis transition state, during asymmetric hydrolysis between the two active sites, or upon product release. These states are currently inaccessible by mutagenesis.

Clamp and ring compartment entrap separate segments of a DNA loop *in vivo*

MukBEF-MatP entraps DNA within its ring and clamp compartments. This implies that in the context of a circular chromosome, DNA would have to enter through one or more entry gates. For biochemical preparation of the cryo-EM sample, however, any loading or partial unloading reactions were bypassed using linear DNA and an ATPase-deficient mutant. As an additional caveat, linear DNA prevents determination of the DNA connectivity that would occur in a physiological context. For example, DNAs in the ring and clamp may originate from the same or from different chromosomes. Hence, we decided to map the DNA binding topology of MukBEF *in vivo*.

We adapted an assay that measures chromosome entrapment by chemically circularized protein complexes for use in *E. coli* (Vazquez Nunez et al., 2019; Wilhelm et al., 2015). In this assay, covalent circularization of a protein compartment around chromosomal DNA preserves DNA association after denaturation of DNA-binding surfaces (Figure 6A). Guided by structural information, we designed cysteine cross-links at the cap (R143C in MukB and Q412C in MukF), the neck (K1246C in MukB and D227C in MukF), and the hinge (C730 and R771C in MukB) to probe DNA entrapment in the ring compartment (Figure 6B and S7A). We also combined cap and neck cysteines with the G67C head cysteine to probe entrapment in the clamp (Figures 6B and S6A). In addition, head and hinge cysteines were combined to probe entrapment in the "frame" compartment, which is the union of ring and clamp.

Combinations of cysteines were introduced into the endogenous *mukFEB* locus, cells were treated with BMOE, and reaction species were identified by SDS-PAGE and in-gel fluorescence (Figure 6C). Cross-linking was specific and efficient at all sites. For some reaction products, species were not completely resolved from each other due to their high molecular weight, identical mass, and mere shape differences. However, depletion of precursors in expected ratios indicated successful multi-site reactions in all cases. For example, the MukB species cross-linked at the heads was reduced from 29% to 12% when combined with the hinge cross-link. This corresponds to a reduction by 60% and is in excellent agreement with the 62% cross-linking efficiency observed for the hinge alone.

First, we tested entrapment in the ring compartment using cap, neck, and hinge cysteine pairs. We treated cells with BMOE, lysed them in agarose plugs to protect chromosomal DNA from shearing, and subjected plugs to electrophoresis in the presence of 0.1% SDS. This denatures and extracts proteins and retains only cross-linked species that have been circularized around DNA. We then digested chromosomal DNA to elute bound proteins. A cross-linked high-molecular-weight MukB species was retained only when cap, neck, and hinge interfaces all contained cysteine pairs, indicating that it is the covalently

CellPress
OPEN ACCESS

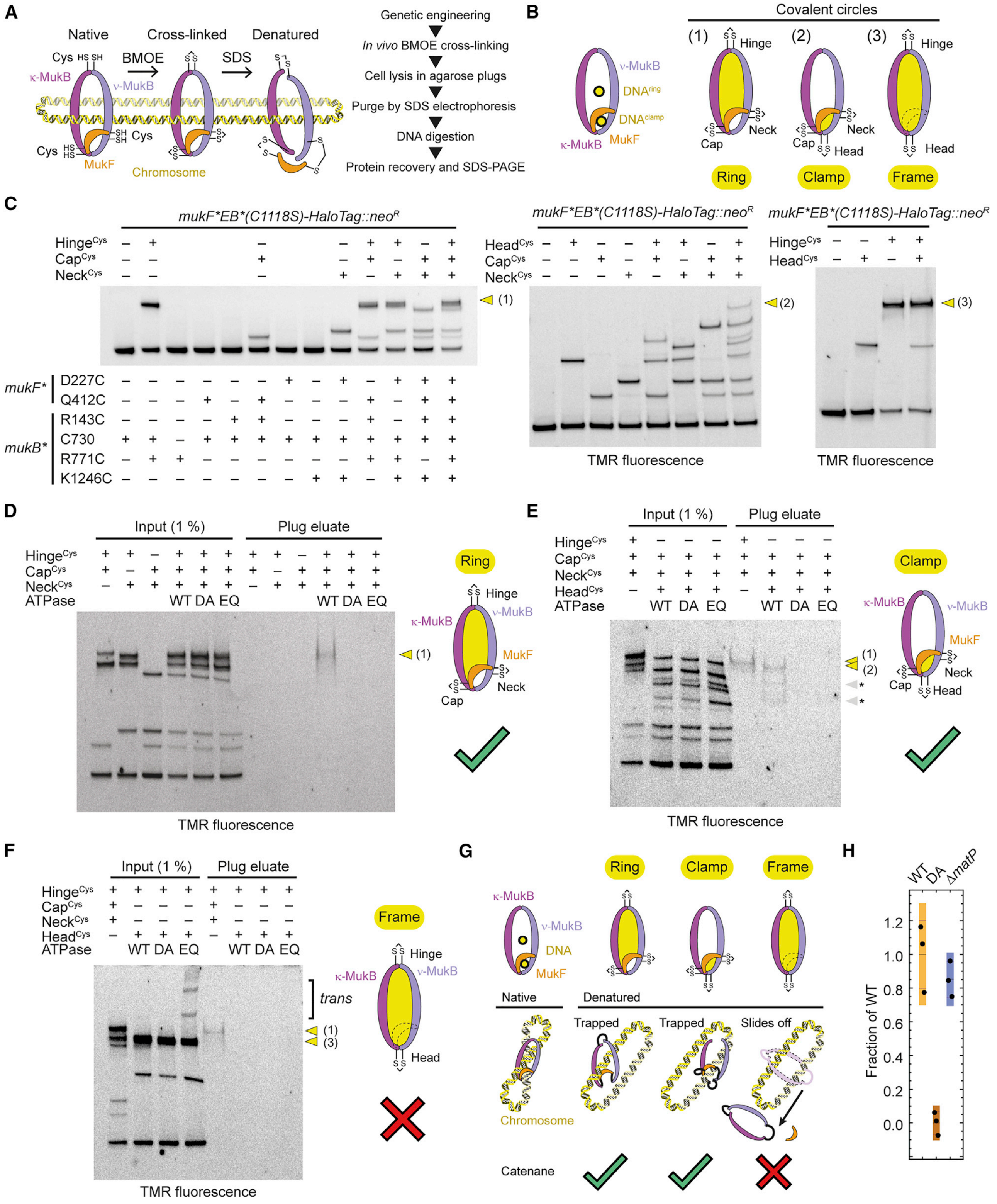

Figure 6. Mapping of DNA binding topology *in vivo*

(A) Principles (left) and workflow (right) of the chromosome entrapment assay in agarose plugs.

(B) Combinations of cross-links used for probing DNA entrapment in the ring, clamp, and frame compartments. Hinge cross-link, C730 and R771C in MukB; cap cross-link, Q412C in MukF and R143C in MukB; neck cross-link, D227C in MukF and K1246C in MukB; head cross-link, G67C in MukB (Figures S6A and S7A).

(legend continued on next page)

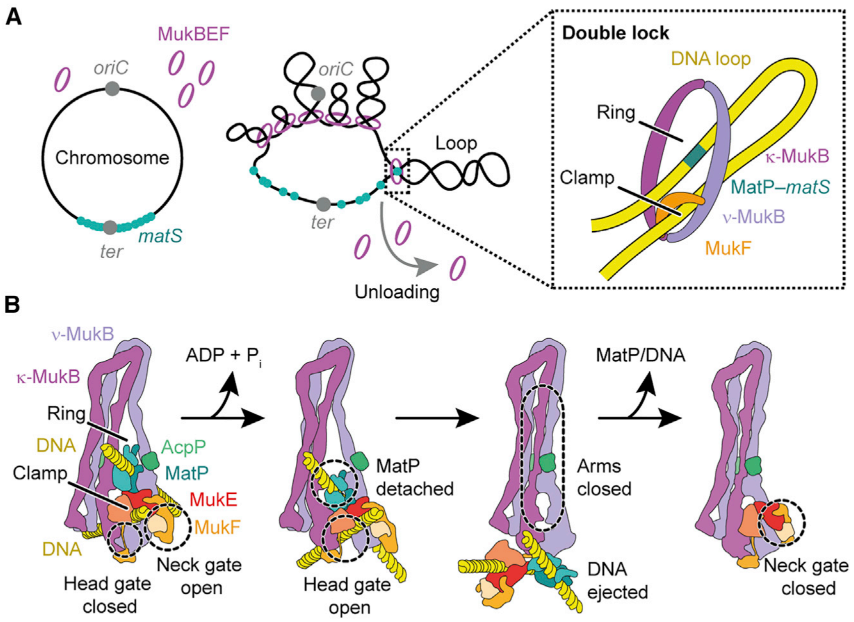

Figure 7. Model for DNA binding and unloading at *matS* sites

(A) Schematic for association of MukBEF with MatP-*matS* in the *Ter* macrodomain. MukBEF organizes the chromosome into loops. Upon invasion of *Ter*, MukBEF encounters MatP-*matS* and unloads via the double-lock topology in the context of a plectonemic loop.

(B) Model for unloading of DNA. A MatP-*matS* encounter is followed by ATP hydrolysis and opening of the head gate to permit exit of the clamped DNA. *matS* DNA follows through neck and head gates, facilitated by the bridge between MatP and MukE. Arm zip-up prevents reversal, and the neck gate closes after MatP/DNA dissociation.

circularized $MukB_2F$ (Figure 6D). The species was not retained from $MukB^{DA}$ or $MukB^{EQ}$ strains, which suggests that DNA entrapment critically depends on ATP hydrolysis.

Next, we tested for DNA entrapment in the clamp compartment using cap, neck, and head cysteines (Figure 6E). The cross-linked MukB species corresponding to the circularized clamp was isolated as the major band, accompanied by smaller amounts of protein that presumably resulted from chemical cross-link reversal during the protein isolation procedure (Shen et al., 2012; Wilhelm and Gruber, 2017). The retained amount was similar to that of the ring compartment species, consistent with the notion that both clamp and ring entrap DNA simultaneously. Protein was not retained from $MukB^{DA}$ or $MukB^{EQ}$ strains. This finding indicates that DNA entrapment in the clamp, as in the ring, depends on ATP hydrolysis *in vivo*.

Next, we tested for DNA entrapment in the frame compartment using the combination of head and hinge cysteines (Figure 6F). Head and hinge cross-linked MukB was not detected in eluates from WT, $MukB^{DA}$, or $MukB^{EQ}$ strains. This and the above two findings are best explained by the notion that the ring and clamp each entrap different strands of the same loop. Cross-linking the frame around this loop allows DNA to slip out of the covalent protein circle because no protein-DNA catenane is formed (Figure 6G).

Taken together, these results fully support entrapment of a loop as suggested by the structure (Figures 1H, 6G, S7B, and S7C). Because no DNA entrapment is detected in the frame compartment and signals for the clamp and ring are similar, most, if not all, complexes with DNA in the clamp must have DNA catenated with the ring, and vice versa. The results are incompatible with entrapment in only ring or clamp, with entrapment of sister chromosomes, or with a loop axis running parallel to the plane of the ring (Figure S7C). Any of these forms would lead to catenation with the frame compartment.

Finally, we investigated whether chromosome entrapment was dependent on MatP. Deletion of the *matP* gene had little, if any, effect on DNA inside the MukBEF ring (Figure 6H). We suggest that DNA entrapment is a more general feature of MukBEF and does not exclusively occur during unloading at *matS* sites.

DISCUSSION

The double-locked loop

The structure of MukBEF bound to MatP-*matS* revealed the simultaneous entrapment of two DNA double helices, topologically separated into ring and clamp compartments. We name this configuration the "double lock" (Figure 7A). The DNA crossing angle in the MatP-bound double lock indicates that a

(C) Combinatorial cross-linking for identification of reaction species. Combinations: hinge, cap, and neck cross-links (left); cap, neck, and head cross-links (middle); head and hinge (right). C730 was mutated to serine when indicated by a minus sign. Cells were grown to stationary phase. Detection as in Figure 5B.
(D) Chromosome entrapment in MukBEF with a covalently closed ring compartment. Input and agarose plug eluate are shown. Detection as in (C). The circular species is retained only in WT ATPase cells. DA, D1406A (blocks ATP binding); EQ, E1407Q (blocks ATP hydrolysis). ATPase is WT if not indicated otherwise.
(E) Chromosome entrapment in MukBEF with covalently closed clamp compartment. As in (D). Species that have undergone chemical cross-link reversal during sample preparation are marked with asterisks.
(F) Chromosome entrapment in MukB with covalently closed frame compartment. As in (D). Species produced by higher-order oligomers in EQ mutants (*trans* cross-links) are indicated.
(G) Structure-based topological interpretation of the entrapment reactions. Only the frame species can slide off DNA because it does not form a protein/DNA catenane.
(H) DNA entrapment in the ring compartment in the absence of MatP. Signal of the plug eluate relative to WT is shown for biological triplicates. Black lines indicate means, purple lines indicate standard deviations, and colored bars indicate 95% credible intervals.
See also Figures S6 and S7.

Table 1. Cryo-EM data collection and model statistics

	Head module EMD-12656 PDB 7NYW	Holocomplex EMD-12657 PDB 7NYX	Holocomplex (apo) EMD-12658 PDB 7NYY	Holocomplex (partially open) EMD-12658 PDB 7NYZ	Holocomplex (open) EMD-12658 PDB 7NZ0	Tetrad EMD-12662 PDB 7NZ2	Tetrad (apposed) EMD-12663 PDB 7NZ3	Dimer (apo) EMD-12664 PDB 7NZ4
Data collection and processing								
Magnification	81,000							
Voltage (kV)	300							
Electron fluence (e^-/Å^2)	40							
Defocus range (μm)	−1 to −3							
Pixel size (Å)	1.07							
Symmetry imposed	C1							
Initial particle images (no.)	3,391,688							
Final particle images (no.)	200,438	74,064	96,150	41,109	60,245	12,010	8,561	4,197
Map resolution (Å)	3.1	4.6	6.8	6.5	6.3	11	11	13
FSC threshold	0.143	0.143	0.143	0.143	0.143	0.143	0.143	0.143
Model								
Initial model used (PDB code)	3EUJ, 3EUH, 3VEA, 3IBP, 6DFL	3IBP, 6H2X, 7NYW	7NYX	7NYX	7NYX	7NYX	7NYX	7NYY
Model resolution (Å)	3.25	5.0	7.5	8.1	7.3	—	—	—
FSC threshold	0.5	0.5	0.5	0.5	0.5			
Map sharpening B factor (Å^2)	−33	−87	−174	−162	−157	—	—	—
Model composition								
Non-hydrogen atoms	24,792	36,100	31,563	36,100	36,100	148,563	74,338	63,192
Protein residues	2,795	4,186	3,910	4,186	4,186	16,752	8372	7824
Nucleic acid residues	104	104	—	104	104	614	312	—
Ligands	PNS: 2	PNS: 2	PNS: 2	PNS: 2	PNS: 2	PNS: 8	PNS: 4	PNS: 4
	ATP: 2	ATP: 2		ATP: 2	ATP: 2	ATP: 8	ATP: 4	
	Mg: 2	Mg: 2		Mg: 2	Mg: 2	Mg: 8	Mg: 4	
RMSDs								
Bond lengths (Å)	0.004	0.004	0.004	0.009	0.005	0.004	0.004	0.005
Bond angles (°)	0.867	0.926	0.942	1.102	0.969	0.929	0.925	0.954
Validation								
MolProbity score	1.57	1.78	1.99	1.77	1.82	1.85	1.83	2.01
Clashscore	5.9	10.9	14.51	10.63	12.31	12.96	12.37	15.24
Poor rotamers (%)	0	0	0	0.08	0.03	0.04	0	0.03
Ramachandran plot								
Favored (%)	96.31	96.57	95.35	96.54	96.64	96.55	96.58	95.31
Allowed (%)	3.69	3.43	4.65	3.46	3.36	3.45	3.42	4.68
Disallowed (%)	0	0	0	0	0	0	0	0.01

DNA loop passes through MukBEF with the loop long axis perpendicular to the ring plane. Topological mapping *in vivo* supports this notion.

DNA entrapment in the MukBEF ring occurs mainly outside of the MatP-*matS* context, as it is unperturbed in Δ*matP* cells. Because localization of MukBEF to chromosomal foci is also largely independent of MatP, and focal MukBEF turns over in an ATP-hydrolysis-dependent manner (Badrinarayanan et al., 2012; Mäkelä and Sherratt, 2020b; Nolivos et al., 2016), it is conceivable that unloading via the double lock happens throughout the chromosome and is not exclusive for the *Ter* region. At *matS* sites, MatP may enhance the positioning of DNA within the ring for an efficient unloading reaction.

How abundant is the double lock? The fraction of cross-linked species retained by the entrapment assay is low, raising the possibility that the double lock is a sparsely populated state. However, the relative signal of the assay will depend on factors such as preservation of chromosomal DNA and the fraction of MukBEF complexes that is loaded. The assay, therefore, likely underestimates abundance by an unknown and possibly large factor and may not be suitable for its quantification. In other words, the data support the existence of the double lock but do not necessarily reveal its incidence. What the experiments do suggest, however, is that a large fraction of MukBEF with a loaded clamp is in the double-lock configuration. Hence, transactions that involve DNA binding inside the clamp may largely progress via the double lock. The same statement applies to DNA entrapment inside the ring. DNA transactions that involve catenation with the ring may predominantly progress via the double lock.

The role of MukBEF dimerization

The architecture of the MukBEF dimer permits binding of two double-locked loops, whereby the loop axes are parallel and monomers are arranged side by side. If loop extrusion by MukBEF monomers was an asymmetric process, similar to what has been observed for monomeric condensin (Ganji et al., 2018), such side-by-side coupling could symmetrize the overall extrusion process. Highly asymmetric loop extrusion is considered a hindrance for physiological chromosome folding and may be resolved using a dimerization mechanism (Banigan and Mirny, 2019). We suggest that the arrangement of the MukBEF dimer is well suited to implement symmetric loop extrusion.

DNA entrapment in MukBEF and other SMC complexes

MukBEF monomers do not entrap sister DNAs or loops with a long axis perpendicular to the one proposed (Figure S7C). However, the low sensitivity of our assay may have precluded detection of rare species. Our experiments also do not exclude formation of "pseudo-topological" loops or "non-topological" loops, which may form in addition to the double lock. The former are established by threading DNA through the same compartment twice, constituting a protein-DNA rotaxane instead of a catenane, whereas the latter do not thread through the complex at all but bind to its outer surface (Figure S7C). These structures may be involved in loop extrusion by cohesin (Davidson et al., 2019; Pradhan et al., 2021; Srinivasan et al., 2018), but their binding by MukBEF is hypothetical. If "pseudo-topological" loops exist, however, they will need to be accommodated in the ring and not in the clamp due to space limitations (Figure S7B). Whereas the clamp is highly constrained by a short and compact MukEF and can thus accommodate only a single DNA, the ring would be able to embrace multiple DNAs and could even enlarge its capacity by extending the elbow and fully opening the arms. How MukBEF would achieve "non-topological" loop extrusion with only a single known biochemical DNA binding site—namely, the clamp—is unclear. A quantitative translocation model, which involves a capacity change of the ring and proceeds via the double-locked loop as a reaction intermediate, has been proposed recently (Marko et al., 2019; Nomidis et al., 2021).

Cysteine cross-linking has been used to map DNA entrapment in other SMC complexes (Chapard et al., 2019; Gligoris et al., 2014; Haering et al., 2008; Vazquez Nunez et al., 2019; Wilhelm et al., 2015). Cross-linking at cap, neck, and hinge of cohesin and Smc-ScpAB (also designated as "SK" cross-links) retains either complex on DNA after denaturation. When cross-linked at cap, neck, and juxtaposed heads (also designated as "JK" cross-links), DNA association is also maintained. Entrapment is not observed when cysteines in hinge and juxtaposed heads are combined (also designated as "JS" cross-links). These patterns are topologically equivalent to the ones determined here. Hence, the MukBEF structure may provide an attractive interpretation for these observations, namely, that cohesin and Smc-ScpAB can entrap DNA in a manner similar to the double-lock configuration.

Interestingly, the head cross-links used for cohesin and Smc-ScpAB can capture a "head-juxtaposed" state, which is different from engaged heads in the corresponding ATP hydrolysis mutants. A large fraction of MukBEF heads are disengaged *in vivo* as judged by our cross-linking experiments, and the G67C cross-link at the heads used for topology mapping can capture the disengaged state. It is, therefore, possible that DNA may be retained in the clamp even after ATP hydrolysis.

Implications for chromosomal turnover of MukBEF

Both ring and clamp of MukBEF fully encircle DNA. This raises the question of how DNA enters and exits these compartments. The structure of MukBEF-MatP-*matS* shows a state before DNA release, whereas the apo structure shows the state after release. The latter also represents the state before DNA has entered the complex. Models for chromosomal turnover of MukBEF will have to fit these observations.

We envision that unloading of *matS* from the ring is coupled to unloading of DNA from the clamp (Figure 7B). For the clamped DNA, the interface between the heads is a prime candidate for an exit gate because its formation is regulated by ATP binding and hydrolysis. Upon ATP hydrolysis and phosphate/nucleotide release, heads disengage and dissociate their DNA binding surfaces. Clamped DNA may then be able to exit via the cleft between disengaging heads. For this to happen, the MukF linker, which also seals the head gate, needs to detach from the ν-MukB head. MukEF can stay bound to and move along with the DNA because the neck gate is open, which allows MukEF to reposition in relation to the ν-MukB head. Concurrently, a deformation at the joints releases MatP, which stays associated with MukEF via the MatP-MukE bridge. Next, the head proximal

arms of MukB zip up and occlude the binding sites for MatP and DNA at the joints and heads, respectively. Release of energy stored as deformations in the hinge-proximal arm will reinforce this process. MatP and DNA are ejected and are free to dissociate from the complex. Finally, closure of the neck gate reverts MukBEF to its apo form. We note that the proposed mechanism would not strictly depend on MatP-*matS* but could also eject "free" DNA from the ring compartment. Its driving force comes from relaxation of MukB into the apo conformation, whereas MatP is a structural element that ensures ideal positioning of DNA close to the exit gate.

The proposed unloading model, which is purely based on structural data, is attractive for several reasons. First, it explains how the process is regulated by ATP hydrolysis, namely, by opening the head gate and blocking the binding sites for MatP and DNA. Second, it explains how the process is enhanced at *matS* sites. Third, the model designates the neck gate as the topological exit gate of the tripartite ring. The equivalent interface in the distantly related cohesin is the exit gate of this complex (Chan et al., 2012; Muir et al., 2020; Murayama and Uhlmann, 2015). We suggest that unloading via the neck gate is a widely conserved activity of SMC complexes.

An interaction of MatP with the MukB hinge, at least in the absence of DNA, has been reported (Fisher et al., 2021; Nolivos et al., 2016) but is not seen in our structures. It is conceivable that this occurs at a different stage during unloading and could suggest subunit and DNA transport within the complex. Interestingly, $MukB^{EQ}$ can associate with *matS* in cells (Nolivos et al., 2016), but DNA entrapment is not detected in our assay. This may point toward a state that binds MatP but does not entrap DNA.

In the light of our structures, DNA entry into MukBEF appears enigmatic, as is the case for other SMC complexes. The joint, which has emerged as a central region regulated by the ATPase, is required for recruitment of Smc-ScpAB to its loading factor, ParB (Gruber and Errington, 2009; Minnen et al., 2016). ParB is typically not present in bacteria that use MukBEF, and no substitute loading factor has been identified. Importantly, loading of MukBEF does not depend on MatP, and a reverse and MatP-independent version of the unloading mechanism proposed above would have to work against a large entropic barrier. This rather unlikely pathway would also depend on ATP binding only, whereas loading *in vivo* requires nucleotide hydrolysis. We suspect that chromosomal loading and unloading of MukBEF are achieved by considerably different means.

Outlook

Here, we have determined the structure of MukBEF both in its apo state and in a MatP/DNA/ATP-bound form, providing molecular insight into how MukBEF is released from chromosomes. This led to the finding that MukBEF can entrap DNA loops in a double-lock configuration, which links two important topological concepts: DNA entrapment inside the ring and inside the clamp compartment. Our work opens the questions of how the double-locked loop is established and how MukBEF operates on this and possibly other types of DNA structures. We anticipate that biochemical reconstitution of the process by which MukBEF organizes chromosomes, coupled to structural analysis, will further our understanding of chromosome folding in bacteria and beyond.

Limitations of the study

Our cryo-EM single-particle analysis may have missed conformational states that do not average well due to heterogeneity or that align poorly against the reference model. Although cross-linking suggests that arm folding of MukBEF does not change upon ATP binding *in vivo*, we note that the ATPase mutants employed do not load onto chromosomal DNA. These experiments, thus, do not resolve whether arm folding may change upon ATP binding while DNA is entrapped.

STAR★METHODS

Detailed methods are provided in the online version of this paper and include the following:

- KEY RESOURCES TABLE
- RESOURCE AVAILABILITY
  - Lead contact
  - Materials availability
  - Data and code availability
- EXPERIMENTAL MODEL AND SUBJECT DETAILS
  - *E. coli* strains
- METHOD DETAILS
  - Protein production and purification
  - *matS* sites and electrophoretic mobility shift assay (EMSA)
  - Cryo-EM sample preparation
  - Cryo-EM data collection
  - Genome engineering
  - *In vivo* cross-linking
  - Chromosome entrapment assay
- QUANTIFICATION AND STATISTICAL ANALYSIS
  - Cryo-EM data analysis
  - Structural model building
  - Analysis of EMSA experiments
  - Analysis of cross-linking experiments
  - Analysis of chromosome entrapment assays

SUPPLEMENTAL INFORMATION

Supplemental information can be found online at https://doi.org/10.1016/j.molcel.2021.10.011.

ACKNOWLEDGMENTS

We thank J. Prince, G. Fisher, and D. Sherratt (Oxford, UK) for discussions and sharing of unpublished results; R. Vazquez Nuñez and S. Gruber (Lausanne, Switzerland) for discussions and advice on the chromosome entrapment assay; all members of the Löwe and K. Nasmyth (Oxford, UK) groups for discussions; D. Komander (WEHI, Australia) for the gift of the GST-hSENP1 expression vector; G. Cannone and all members of the LMB electron microscopy facility for excellent EM training and support; and T. Darling and J. Grimmett (LMB scientific computing) for computing support. F.B. was supported by an EMBO Advanced fellowship (ALTF 605-2019). This work was funded by the UKRI Medical Research Council.

AUTHOR CONTRIBUTIONS

F.B. performed protein purification, cryo-EM sample preparation, cryo-EM data acquisition and analysis, model building, strain construction, and all other experiments; L.F.H.F. designed the genome mutagenesis strategy; F.B. adapted this strategy for combinatorial mutagenesis; J.W.C. supervised technology development; J.L. supervised the overall study; F.B. prepared the manuscript with contributions from all authors.

DECLARATION OF INTERESTS

The authors declare no competing interests.

Received: June 29, 2021
Revised: August 31, 2021
Accepted: October 12, 2021
Published: November 4, 2021

REFERENCES

Afonine, P.V., Poon, B.K., Read, R.J., Sobolev, O.V., Terwilliger, T.C., Urzhumtsev, A., and Adams, P.D. (2018). Real-space refinement in PHENIX for cryo-EM and crystallography. Acta Crystallogr. D Struct. Biol. *74*, 531–544.

Badrinarayanan, A., Reyes-Lamothe, R., Uphoff, S., Leake, M.C., and Sherratt, D.J. (2012). In vivo architecture and action of bacterial structural maintenance of chromosome proteins. Science *338*, 528–531.

Banigan, E.J., and Mirny, L.A. (2019). Limits of Chromosome Compaction by Loop-Extruding Motors. Phys. Rev. X *9*, 031007.

Bürmann, F., Shin, H.-C., Basquin, J., Soh, Y.-M., Giménez-Oya, V., Kim, Y.-G., Oh, B.-H., and Gruber, S. (2013). An asymmetric SMC-kleisin bridge in prokaryotic condensin. Nat. Struct. Mol. Biol. *20*, 371–379.

Bürmann, F., Lee, B.-G., Than, T., Sinn, L., O'Reilly, F.J., Yatskevich, S., Rappsilber, J., Hu, B., Nasmyth, K., and Löwe, J. (2019). A folded conformation of MukBEF and cohesin. Nat. Struct. Mol. Biol. *26*, 227–236.

Butt, T.R., Edavettal, S.C., Hall, J.P., and Mattern, M.R. (2005). SUMO fusion technology for difficult-to-express proteins. Protein Expr. Purif. *43*, 1–9.

Chan, K.-L., Roig, M.B., Hu, B., Beckouët, F., Metson, J., and Nasmyth, K. (2012). Cohesin's DNA exit gate is distinct from its entrance gate and is regulated by acetylation. Cell *150*, 961–974.

Chapard, C., Jones, R., van Oepen, T., Scheinost, J.C., and Nasmyth, K. (2019). Sister DNA Entrapment between Juxtaposed Smc Heads and Kleisin of the Cohesin Complex. Mol. Cell *75*, 224–237.e5.

Collier, J.E., Lee, B.-G., Roig, M.B., Yatskevich, S., Petela, N.J., Metson, J., Voulgaris, M., Gonzalez Llamazares, A., Löwe, J., and Nasmyth, K.A. (2020). Transport of DNA within cohesin involves clamping on top of engaged heads by Scc2 and entrapment within the ring by Scc3. eLife *9*, e59560.

Croll, T.I. (2018). ISOLDE: a physically realistic environment for model building into low-resolution electron-density maps. Acta Crystallogr. D Struct. Biol. *74*, 519–530.

Cronan, J.E. (2014). The chain-flipping mechanism of ACP (acyl carrier protein)-dependent enzymes appears universal. Biochem. J. *460*, 157–163.

Cuylen, S., Metz, J., and Haering, C.H. (2011). Condensin structures chromosomal DNA through topological links. Nat. Struct. Mol. Biol. *18*, 894–901.

Danilova, O., Reyes-Lamothe, R., Pinskaya, M., Sherratt, D., and Possoz, C. (2007). MukB colocalizes with the oriC region and is required for organization of the two Escherichia coli chromosome arms into separate cell halves. Mol. Microbiol. *65*, 1485–1492.

Davidson, I.F., and Peters, J.-M. (2021). Genome folding through loop extrusion by SMC complexes. Nat. Rev. Mol. Cell Biol. *22*, 445–464.

Davidson, I.F., Bauer, B., Goetz, D., Tang, W., Wutz, G., and Peters, J.-M. (2019). DNA loop extrusion by human cohesin. Science *366*, 1338–1345.

Diebold-Durand, M.-L., Lee, H., Ruiz Avila, L.B., Noh, H., Shin, H.-C., Im, H., Bock, F.P., Bürmann, F., Durand, A., Basfeld, A., et al. (2017). Structure of Full-Length SMC and Rearrangements Required for Chromosome Organization. Mol. Cell *67*, 334–347.e5.

Dupaigne, P., Tonthat, N.K., Espéli, O., Whitfill, T., Boccard, F., and Schumacher, M.A. (2012). Molecular basis for a protein-mediated DNA-bridging mechanism that functions in condensation of the E. coli chromosome. Mol. Cell *48*, 560–571.

Emsley, P., Lohkamp, B., Scott, W.G., and Cowtan, K. (2010). Features and development of Coot. Acta Crystallogr. D Biol. Crystallogr. *66*, 486–501.

Engler, C., Kandzia, R., and Marillonnet, S. (2008). A one pot, one step, precision cloning method with high throughput capability. PLoS ONE *3*, e3647.

Fennell-Fezzie, R., Gradia, S.D., Akey, D., and Berger, J.M. (2005). The MukF subunit of Escherichia coli condensin: architecture and functional relationship to kleisins. EMBO J. *24*, 1921–1930.

Fisher, G.L.M., Bolla, J.R., Rajasekar, K.V., Mäkelä, J., Baker, R., Zhou, M., Prince, J.P., Stracy, M., Robinson, C.V., Arciszewska, L.K., et al. (2021). Competitive binding of MatP and topoisomerase IV to the MukB hinge domain. eLife *10*, e70444.

Fredens, J., Wang, K., de la Torre, D., Funke, L.F.H., Robertson, W.E., Christova, Y., Chia, T., Schmied, W.H., Dunkelmann, D.L., Beránek, V., et al. (2019). Total synthesis of Escherichia coli with a recoded genome. Nature *569*, 514–518.

Ganji, M., Shaltiel, I.A., Bisht, S., Kim, E., Kalichava, A., Haering, C.H., and Dekker, C. (2018). Real-time imaging of DNA loop extrusion by condensin. Science *360*, 102–105.

Gligoris, T.G., Scheinost, J.C., Bürmann, F., Petela, N., Chan, K.-L., Uluocak, P., Beckouët, F., Gruber, S., Nasmyth, K., and Löwe, J. (2014). Closing the cohesin ring: structure and function of its Smc3-kleisin interface. Science *346*, 963–967.

Gloyd, M., Ghirlando, R., and Guarné, A. (2011). The role of MukE in assembling a functional MukBEF complex. J. Mol. Biol. *412*, 578–590.

Gruber, S., and Errington, J. (2009). Recruitment of condensin to replication origin regions by ParB/SpoOJ promotes chromosome segregation in B. subtilis. Cell *137*, 685–696.

Gully, D., Moinier, D., Loiseau, L., and Bouveret, E. (2003). New partners of acyl carrier protein detected in Escherichia coli by tandem affinity purification. FEBS Lett. *548*, 90–96.

Haering, C.H., and Gruber, S. (2016). SnapShot: SMC protein complexes part I. Cell *164*, 326–326.e1.

Haering, C.H., Schoffnegger, D., Nishino, T., Helmhart, W., Nasmyth, K., and Löwe, J. (2004). Structure and stability of cohesin's Smc1-kleisin interaction. Mol. Cell *15*, 951–964.

Haering, C.H., Farcas, A.-M., Arumugam, P., Metson, J., and Nasmyth, K. (2008). The cohesin ring concatenates sister DNA molecules. Nature *454*, 297–301.

Hassler, M., Shaltiel, I.A., and Haering, C.H. (2018). Towards a Unified Model of SMC Complex Function. Curr. Biol. *28*, R1266–R1281.

Hassler, M., Shaltiel, I.A., Kschonsak, M., Simon, B., Merkel, F., Thärichen, L., Bailey, H.J., Macošek, J., Bravo, S., Metz, J., et al. (2019). Structural Basis of an Asymmetric Condensin ATPase Cycle. Mol. Cell *74*, 1175–1188.e9.

Higashi, T.L., Eickhoff, P., Sousa, J.S., Locke, J., Nans, A., Flynn, H.R., Snijders, A.P., Papageorgiou, G., O'Reilly, N., Chen, Z.A., et al. (2020). A Structure-Based Mechanism for DNA Entry into the Cohesin Ring. Mol. Cell *79*, 917–933.e9.

Hiraga, S., Niki, H., Ogura, T., Ichinose, C., Mori, H., Ezaki, B., and Jaffé, A. (1989). Chromosome partitioning in Escherichia coli: novel mutants producing anucleate cells. J. Bacteriol. *171*, 1496–1505.

Hopfner, K.P., Karcher, A., Shin, D.S., Craig, L., Arthur, L.M., Carney, J.P., and Tainer, J.A. (2000). Structural biology of Rad50 ATPase: ATP-driven conformational control in DNA double-strand break repair and the ABC-ATPase superfamily. Cell *101*, 789–800.

Inoue, H., Nojima, H., and Okayama, H. (1990). High efficiency transformation of Escherichia coli with plasmids. Gene *96*, 23–28.

CellPress
OPEN ACCESS

Ivanov, D., and Nasmyth, K. (2005). A topological interaction between cohesin rings and a circular minichromosome. Cell *122*, 849–860.

Kanno, T., Berta, D.G., and Sjögren, C. (2015). The Smc5/6 Complex Is an ATP-Dependent Intermolecular DNA Linker. Cell Rep. *12*, 1471–1482.

Karaboja, X., Ren, Z., Brandão, H.B., Paul, P., Rudner, D.Z., and Wang, X. (2021). XerD unloads bacterial SMC complexes at the replication terminus. Mol. Cell *81*, 756–766.e8.

Käshammer, L., Saathoff, J.-H., Lammens, K., Gut, F., Bartho, J., Alt, A., Kessler, B., and Hopfner, K.-P. (2019). Mechanism of DNA End Sensing and Processing by the Mre11-Rad50 Complex. Mol. Cell *76*, 382–394.e6.

Kreamer, N.N.K., Chopra, R., Caughlan, R.E., Fabbro, D., Fang, E., Gee, P., Hunt, I., Li, M., Leon, B.C., Muller, L., et al. (2018). Acylated-acyl carrier protein stabilizes the Pseudomonas aeruginosa WaaP lipopolysaccharide heptose kinase. Sci. Rep. *8*, 14124.

Lee, B.-G., Merkel, F., Allegretti, M., Hassler, M., Cawood, C., Lecomte, L., O'Reilly, F.J., Sinn, L.R., Gutierrez-Escribano, P., Kschonsak, M., et al. (2020). Cryo-EM structures of holo condensin reveal a subunit flip-flop mechanism. Nat. Struct. Mol. Biol. *27*, 743–751.

Li, Y., Schoeffler, A.J., Berger, J.M., and Oakley, M.G. (2010). The crystal structure of the hinge domain of the Escherichia coli structural maintenance of chromosomes protein MukB. J. Mol. Biol. *395*, 11–19.

Lioy, V.S., Cournac, A., Marbouty, M., Duigou, S., Mozziconacci, J., Espéli, O., Boccard, F., and Koszul, R. (2018). Multiscale Structuring of the E. coli Chromosome by Nucleoid-Associated and Condensin Proteins. Cell *172*, 771–783.e18.

Lobry, J.R. (1996). Asymmetric substitution patterns in the two DNA strands of bacteria. Mol. Biol. Evol. *13*, 660–665.

Mäkelä, J., and Sherratt, D. (2020a). SMC complexes organize the bacterial chromosome by lengthwise compaction. Curr. Genet. *66*, 895–899.

Mäkelä, J., and Sherratt, D.J. (2020b). Organization of the Escherichia coli Chromosome by a MukBEF Axial Core. Mol. Cell *78*, 250–260.e5.

Marko, J.F., De Los Rios, P., Barducci, A., and Gruber, S. (2019). DNA-segment-capture model for loop extrusion by structural maintenance of chromosome (SMC) protein complexes. Nucleic Acids Res. *47*, 6956–6972.

Mastronarde, D.N. (2005). Automated electron microscope tomography using robust prediction of specimen movements. J. Struct. Biol. *152*, 36–51.

Matoba, K., Yamazoe, M., Mayanagi, K., Morikawa, K., and Hiraga, S. (2005). Comparison of MukB homodimer versus MukBEF complex molecular architectures by electron microscopy reveals a higher-order multimerization. Biochem. Biophys. Res. Commun. *333*, 694–702.

Mercier, R., Petit, M.-A., Schbath, S., Robin, S., El Karoui, M., Boccard, F., and Espéli, O. (2008). The MatP/matS site-specific system organizes the terminus region of the E. coli chromosome into a macrodomain. Cell *135*, 475–485.

Minnen, A., Bürmann, F., Wilhelm, L., Anchimiuk, A., Diebold-Durand, M.-L., and Gruber, S. (2016). Control of Smc Coiled Coil Architecture by the ATPase Heads Facilitates Targeting to Chromosomal ParB/parS and Release onto Flanking DNA. Cell Rep. *14*, 2003–2016.

Miyazaki, K. (2015). Molecular engineering of a PheS counterselection marker for improved operating efficiency in Escherichia coli. Biotechniques *58*, 86–88.

Muir, K.W., Li, Y., Weis, F., and Panne, D. (2020). The structure of the cohesin ATPase elucidates the mechanism of SMC-kleisin ring opening. Nat. Struct. Mol. Biol. *27*, 233–239.

Murayama, Y., and Uhlmann, F. (2014). Biochemical reconstitution of topological DNA binding by the cohesin ring. Nature *505*, 367–371.

Murayama, Y., and Uhlmann, F. (2015). DNA Entry into and Exit out of the Cohesin Ring by an Interlocking Gate Mechanism. Cell *163*, 1628–1640.

Nasmyth, K. (2001). Disseminating the genome: joining, resolving, and separating sister chromatids during mitosis and meiosis. Annu. Rev. Genet. *35*, 673–745.

Niki, H., and Yano, K. (2016). In vitro topological loading of bacterial condensin MukB on DNA, preferentially single-stranded DNA rather than double-stranded DNA. Sci. Rep. *6*, 29469.

Niki, H., Imamura, R., Kitaoka, M., Yamanaka, K., Ogura, T., and Hiraga, S. (1992). E.coli MukB protein involved in chromosome partition forms a homodimer with a rod-and-hinge structure having DNA binding and ATP/GTP binding activities. EMBO J. *11*, 5101–5109.

Nolivos, S., Upton, A.L., Badrinarayanan, A., Müller, J., Zawadzka, K., Wiktor, J., Gill, A., Arciszewska, L., Nicolas, E., and Sherratt, D. (2016). MatP regulates the coordinated action of topoisomerase IV and MukBEF in chromosome segregation. Nat. Commun. *7*, 10466.

Nomidis, S.K., Carlon, E., Gruber, S., and Marko, J.F. (2021). DNA tension-modulated translocation and loop extrusion by SMC complexes revealed by molecular dynamics simulations. bioRxiv. https://doi.org/10.1101/2021.03.15.435506.

Palecek, J.J., and Gruber, S. (2015). Kite Proteins: a Superfamily of SMC/Kleisin Partners Conserved Across Bacteria, Archaea, and Eukaryotes. Structure *23*, 2183–2190.

Pettersen, E.F., Goddard, T.D., Huang, C.C., Meng, E.C., Couch, G.S., Croll, T.I., Morris, J.H., and Ferrin, T.E. (2021). UCSF ChimeraX: Structure visualization for researchers, educators, and developers. Protein Sci. *30*, 70–82.

Pradhan, B., Barth, R., Kim, E., Davidson, I.F., Bauer, B., van Laar, T., Yang, W., Ryu, J.-K., van der Torre, J., Peters, J.-M., et al. (2021). SMC complexes can traverse physical roadblocks bigger than their ring size. bioRxiv. https://doi.org/10.1101/2021.07.15.452501.

Prince, J.P., Bolla, J.R., Fisher, G.L.M., Mäkelä, J., Robinson, C.V., Arciszewska, L.K., and Sherratt, D.J. (2021). Acyl Carrier Protein is essential for MukBEF action in Escherichia coli chromosome organization-segregation. bioRxiv. https://doi.org/10.1101/2021.04.12.439405.

Rajasekar, K.V., Baker, R., Fisher, G.L.M., Bolla, J.R., Mäkelä, J., Tang, M., Zawadzka, K., Koczy, O., Wagner, F., Robinson, C.V., et al. (2019). Dynamic architecture of the Escherichia coli structural maintenance of chromosomes (SMC) complex, MukBEF. Nucleic Acids Res. *47*, 9696–9707.

Rawdon, E.J., Dorier, J., Racko, D., Millett, K.C., and Stasiak, A. (2016). How topoisomerase IV can efficiently unknot and decatenate negatively supercoiled DNA molecules without causing their torsional relaxation. Nucleic Acids Res. *44*, 4528–4538.

Rohou, A., and Grigorieff, N. (2015). CTFFIND4: Fast and accurate defocus estimation from electron micrographs. J. Struct. Biol. *192*, 216–221.

Rosenthal, P.B., and Henderson, R. (2003). Optimal determination of particle orientation, absolute hand, and contrast loss in single-particle electron cryomicroscopy. J. Mol. Biol. *333*, 721–745.

Russo, C.J., and Passmore, L.A. (2014). Electron microscopy: Ultrastable gold substrates for electron cryomicroscopy. Science *346*, 1377–1380.

Scheres, S.H.W. (2012). A Bayesian view on cryo-EM structure determination. J. Mol. Biol. *415*, 406–418.

Schleiffer, A., Kaitna, S., Maurer-Stroh, S., Glotzer, M., Nasmyth, K., and Eisenhaber, F. (2003). Kleisins: a superfamily of bacterial and eukaryotic SMC protein partners. Mol. Cell *11*, 571–575.

Shen, B.-Q., Xu, K., Liu, L., Raab, H., Bhakta, S., Kenrick, M., Parsons-Reponte, K.L., Tien, J., Yu, S.-F., Mai, E., et al. (2012). Conjugation site modulates the in vivo stability and therapeutic activity of antibody-drug conjugates. Nat. Biotechnol. *30*, 184–189.

Shi, Z., Gao, H., Bai, X.-C., and Yu, H. (2020). Cryo-EM structure of the human cohesin-NIPBL-DNA complex. Science *368*, 1454–1459.

Srinivasan, M., Scheinost, J.C., Petela, N.J., Gligoris, T.G., Wissler, M., Ogushi, S., Collier, J.E., Voulgaris, M., Kurze, A., Chan, K.-L., et al. (2018). The Cohesin Ring Uses Its Hinge to Organize DNA Using Non-topological as well as Topological Mechanisms. Cell *173*, 1508–1519.e18.

Stoddart, J.F. (2009). The chemistry of the mechanical bond. Chem. Soc. Rev. *38*, 1802–1820.

Studier, F.W. (2005). Protein production by auto-induction in high density shaking cultures. Protein Expr. Purif. *41*, 207–234.

Vazquez Nunez, R., Ruiz Avila, L.B., and Gruber, S. (2019). Transient DNA Occupancy of the SMC Interarm Space in Prokaryotic Condensin. Mol. Cell *75*, 209–223.e6.

Vazquez Nunez, R., Polyhach, Y., Soh, Y.-M., Jeschke, G., and Gruber, S. (2021). Gradual opening of Smc arms in prokaryotic condensin. Cell Rep. *35*, 109051.

Wagner, T., Merino, F., Stabrin, M., Moriya, T., Antoni, C., Apelbaum, A., Hagel, P., Sitsel, O., Raisch, T., Prumbaum, D., et al. (2019). SPHIRE-crYOLO is a fast and accurate fully automated particle picker for cryo-EM. Commun. Biol. *2*, 218.

Wang, K., Fredens, J., Brunner, S.F., Kim, S.H., Chia, T., and Chin, J.W. (2016). Defining synonymous codon compression schemes by genome recoding. Nature *539*, 59–64.

Waterhouse, A., Bertoni, M., Bienert, S., Studer, G., Tauriello, G., Gumienny, R., Heer, F.T., de Beer, T.A.P., Rempfer, C., Bordoli, L., et al. (2018). SWISS-MODEL: homology modelling of protein structures and complexes. Nucleic Acids Res. *46*, W296–W303.

Weitzel, C.S., Waldman, V.M., Graham, T.A., and Oakley, M.G. (2011). A repeated coiled-coil interruption in the Escherichia coli condensin MukB. J. Mol. Biol. *414*, 578–595.

Wilhelm, L., and Gruber, S. (2017). A Chromosome Co-Entrapment Assay to Study Topological Protein-DNA Interactions. Methods Mol. Biol. *1624*, 117–126.

Wilhelm, L., Bürmann, F., Minnen, A., Shin, H.-C., Toseland, C.P., Oh, B.-H., and Gruber, S. (2015). SMC condensin entraps chromosomal DNA by an ATP hydrolysis dependent loading mechanism in Bacillus subtilis. eLife *4*, e06659.

Woo, J.-S., Lim, J.-H., Shin, H.-C., Suh, M.-K., Ku, B., Lee, K.-H., Joo, K., Robinson, H., Lee, J., Park, S.-Y., et al. (2009). Structural studies of a bacterial condensin complex reveal ATP-dependent disruption of intersubunit interactions. Cell *136*, 85–96.

Yatskevich, S., Rhodes, J., and Nasmyth, K. (2019). Organization of Chromosomal DNA by SMC Complexes. Annu. Rev. Genet. *53*, 445–482.

Yu, D., Ellis, H.M., Lee, E.C., Jenkins, N.A., Copeland, N.G., and Court, D.L. (2000). An efficient recombination system for chromosome engineering in Escherichia coli. Proc. Natl. Acad. Sci. USA *97*, 5978–5983.

Zawadzka, K., Zawadzki, P., Baker, R., Rajasekar, K.V., Wagner, F., Sherratt, D.J., and Arciszewska, L.K. (2018). MukB ATPases are regulated independently by the N- and C-terminal domains of MukF kleisin. eLife *7*, e31522.

STAR★METHODS

KEY RESOURCES TABLE

REAGENT or RESOURCE	SOURCE	IDENTIFIER
**Chemicals, peptides, and recombinant proteins**		
4-chloro-phenylalanine (4-CP)	Sigma-Aldrich	Cat#C6506-5G
Adenosine triphosphate (ATP)	Sigma-Aldrich	Cat#A26209-10G
Benzonase	Merck	Cat#E1014-25KU
Bis(maleimido)ethane (BMOE)	Thermo Fisher Scientific	Cat#22323
B-PER	Thermo Fisher Scientific	Cat#78266
Costar Spin-X 0.45 μm filter	Corning	Cat#8162
Glutathione Sepharose 4B	GE Healthcare	Cat#17-0756-01
GST-hSENP1	MRC-LMB	N/A
HaloTag TMR ligand	Promega	Cat#G8251
HiPrep 26/60 Sephacryl S-200	GE Healthcare	Cat#17-1195-01
HisTrap HP 5 mL	GE Healthcare	Cat#17-5248-02
HiTrap Heparin HP 5 mL	GE Healthcare	Cat#17-0407-03
HiTrap Q HP 5 mL	GE Healthcare	Cat#17-1154-01
HiTrap SP HP 5 mL	GE Healthcare	Cat#17-1152-01
Low-Melt agarose	BioRad	Cat#1613111
Ni-NTA agarose	QIAGEN	Cat#30210
P. thracensis MatP	This paper	N/A
P. thracensis MukB	This paper	N/A
P. thracensis MukB(E1407Q)	This paper	N/A
P. thracensis $MukB_2E_4F_2$	This paper	N/A
P. thracensis $MukB(E1407Q)_2E_4F_2$	This paper	N/A
Protease inhibitor cocktail (cOmplete, EDTA-free)	Roche	Cat#48047900
ReadyLyse lysozyme	Lucigen	Cat#E0057-D2
Superose 6 Increase 10/300 GL	GE Healthcare	Cat#29-0915-96
Superose 6 Increase 3.2/300	GE Healthcare	Cat#29-0915-98
UltrAuFoil R2/2 Au 200 mesh	Quantifoil	Cat#N1-A1BnAu20-01
Vivaspin 2 MWCO 30	Sartorius	Cat#VS0222
Vivaspin 20 MWCO 10	Sartorius	Cat#VS2002
Vivaspin 20 MWCO 30	Sartorius	Cat#VS2021
Zeba Micro Spin 7K MWCO	Thermo Fisher Scientific	Cat#89877
β-octyl glucoside	Sigma-Aldrich	Cat#O-8001
**Critical commercial assays**		
6% DNA retardation gel	Thermo Fisher Scientific	Cat#EC63655BOX
LDS sample buffer	Thermo Fisher Scientific	Cat#NP0007
NuPAGE 3-8% Tris-acetate gel	Thermo Fisher Scientific	Cat#EA03755BOX
NuPAGE 4-12% Bis-tris gel	Thermo Fisher Scientific	Cat#NP0321BOX
**Deposited data**		
Raw micrographs of MukB(EQ)EF in complex with MatP and DNA	This paper	EMPIAR-10755
Cryo-EM densities, see Table 1	This paper	N/A
Atom coordinates, see Table 1	This paper	N/A
Original gel images	This paper	https://dx.doi.org/10.17632/rvd864rz78.1

(*Continued on next page*)

Continued

REAGENT or RESOURCE	SOURCE	IDENTIFIER
Experimental models: Organisms/strains		
E. coli strains, see Table S1 and Data S1	N/A	N/A
Oligonucleotides		
21 bp matS1 strand 1: [6-FAM]CACTGTGACATTGTCACGGCA	This paper	FBA747
21 bp matS1 strand 2: TGCCGTGACAATGTCACAGTG	This paper	FBA748
21 bp matS2 strand 1: [6-FAM]CACTGTTACAGTGTAACGGCA	This paper	FBA765
21 bp matS2 strand 2, TGCCGTTACACTGTAACAGTG	This paper	FBA766
80 bp matS2 strand 1: CTCGCCTGTAAAGTAGGCATTAGTTGT TCGTAGTGCTCGTCTGGCTCTGGATTACCCGCCACTGTTACA TTGTAACGGCA	This paper	FBA769
80 bp matS2 strand 2: TGCCGTTACAATGTAACAGTGGCGGG TAATCCAGAGCCAGACGAGCACTACGAACAACTAATGCCTA CTTTACAGGCGAG	This paper	FBA770
Recombinant DNA		
Plasmid DNA, see Table S2 and Data S1	N/A	N/A
Software and algorithms		
ChimeraX	Pettersen et al., 2021	https://www.cgl.ucsf.edu/chimerax/
Coot	Emsley et al., 2010	https://www2.mrc-lmb.cam.ac.uk/personal/pemsley/coot/
crYOLO	Wagner et al., 2019	https://cryolo.readthedocs.io/en/stable
CTFFIND4	Rohou and Grigorieff, 2015	https://grigoriefflab.umassmed.edu/ctffind4
ISOLDE	Croll, 2018	https://isolde.cimr.cam.ac.uk/
PHENIX v1.19	Afonine et al., 2018	https://phenix-online.org
RELION v3.1	Scheres, 2012	https://relion.readthedocs.io/en/release-3.1/
SerialEM	Mastronarde, 2005	https://bio3d.colorado.edu/SerialEM/
Wolfram Mathematica	Wolfram Research	https://wolfram.com
Other		
ÄKTA Ettan	GE Healthcare	N/A
GIF imaging filter	Gatan	https://www.gatan.com/products/tem-imaging-spectroscopy
K3 Camera	Gatan	https://www.gatan.com/products/tem-imaging-spectroscopy
SC7620 glow discharger	Quorum	https://www.quorumtech.com/sc7620/
Titan Krios, X-FEG	Thermo Fisher Scientific	https://www.thermofisher.com/us/en/home/electron-microscopy/products/transmission-electron-microscopes.html
Typhoon FLA9000	GE Healthcare	N/A
Vitrobot Mark IV	Thermo Fisher Scientific	https://www.thermofisher.com/us/en/home/electron-microscopy/products/sample-preparation-equipment-em/vitrobot-system.html

RESOURCE AVAILABILITY

Lead contact

Further information and requests for resources and reagents should be directed to and will be fulfilled by the lead contact, Jan Löwe (jyl@mrc-lmb.cam.ac.uk).

Materials availability

All unique reagents generated in this study are available upon request, restricted by the use of a material transfer agreement (MTA).

Data and code availability

- Raw micrographs and particle parameters have been deposited in the EMPIAR. EM density maps have been deposited in the EMDB. Atom coordinates have been deposited in the PDB. Raw gel images have been deposited at Mendeley. The deposited data are available as of the date of publication. Accession numbers are listed in the key resources table. All other data will be shared by the lead contact upon request.
- This paper does not report original code.
- Any additional information required to reanalyze the data reported in this paper is available from the lead contact upon request.

EXPERIMENTAL MODEL AND SUBJECT DETAILS

E. coli strains

Strains are based on *E. coli* MG1655 and are listed in Table S1. The parental strain was obtained from the DSMZ strain collection (DSM 18039). All strains were viable in LB at 37°C, except for Δ*mukB*, *mukB*(S1366R), *mukB*(D1407A) and mukB(E1406Q) derivatives which were cultivated at 22°C, their permissive temperature. The strain containing cap, neck, and head cysteines for circularization of the clamp compartment (SFB202) was viable at 37°C but grew with a reduced rate, whereas all other strains with functional alleles grew with rates similar to WT. Strains were verified by phenotype, marker analysis, PCR, and Sanger sequencing as required. Precultures for all experiments were grown side-by-side to stationary phase and stored at 4°C for up to two weeks. Proteins were purified from *E. coli* BL21-Gold(DE3) or *E. coli* C41(DE3) transformed with the appropriate expression plasmids as indicated (see also Table S2).

METHOD DETAILS

Protein production and purification

All protein concentrations were determined by absorbance at 280 nm using theoretical absorption coefficients. Annotated sequences of expression constructs are provided in Data S1. See also Table S2.

GST-hSENP1

GST-tagged hSENP1 protease was produced from a T7 expression plasmid (pFB83) in *E. coli* C41(DE3) by induction with 1 mM IPTG in 2xYT medium at 18°C overnight. All purification steps were carried out at 4°C. 83 g of cells were resuspended in 300 mL of buffer A (50 mM Tris/HCl pH 8.0 at room temperature (RT), 150 mM NaCl, 1 mM EDTA pH 8.0 at RT, 5% glycerol, 2 mM DTT) supplemented with protease inhibitor cocktail (Roche) and Benzonase (Merck) and lysed at 172 MPa in a high-pressure homogenizer. The lysate was cleared by centrifugation at 40,000 x g for 30 min and incubated with 10 mL Glutathione Sepharose 4B (GE Healthcare) for 14 h. The resin washed with 15 column volumes (CV) of buffer A, 5 CV of buffer B (50 mM Tris/HCl pH 8.0 at RT, 500 mM NaCl, 1 mM EDTA pH 8 at RT, 5% glycerol, 2 mM DTT) and protein was eluted in 5 CV of buffer A containing 3 mg/mL glutathione. Aliquots of the eluate were passed through a 0.22 μm filter and injected into a HiPrep 26/60 Sephacryl S-200 column (GE Healthcare) in buffer G1 (25 mM Tris/HCl pH 8.0 at RT, 250 mM NaCl, 0.5 mM DTT). Peak fractions were pooled, concentrated to 9.3 mg/mL on a Vivaspin 20 MWCO 30 filter (Sartorius), aliquoted, frozen in liquid nitrogen and stored at −80°C.

MukBEF

Wild-type *P. thracensis* MukBEF (NCBI accession identifiers WP_046975681.1, WP_046975682.1, and WP_046975683.1) was produced from a polycistronic expression construct assembled into a pET28 based backbone by Golden Gate cloning (Engler et al., 2008) (pFB403). The construct contained a His_6-SUMO tag fused to residue 1 of MukB which allowed affinity purification and scar-less tag removal by hSENP1 protease (Butt et al., 2005). The complex was produced in *E. coli* BL21-Gold(DE3) by autoinduction in ZYP-5052 media (Studier, 2005) at 24°C. All purification steps were carried out at 4°C. 15 g of cells were resuspended in 90 mL of IMAC buffer (50 mM Tris, 300 mM NaCl, 40 mM imidazole, 1 mM TCEP, pH 7.4 at RT) supplemented with protease inhibitor cocktail and Benzonase and lysed at 172 MPa in a high-pressure homogenizer. The lysate was cleared by centrifugation at 96,000 x g for 30 min, passed through a 0.45 μm filter, and incubated for 30 min with 25 mL Ni-NTA agarose (QIAGEN) equilibrated in IMAC buffer. The resin was packed into a gravity flow column and washed with 3 × 50 mL IMAC buffer, then resuspended in 25 mL IMAC buffer containing 1 mg GST-hSENP1 and incubated for 1 h on a roller. Eluate was collected and pooled with a 12.5 mL wash using IMAC buffer, diluted with 18.8 mL buffer Q (10 mM Tris, pH 7.4 at RT), passed through a 0.22 μm filter and applied to a 20 mL HiTrap Heparin HP column (GE Healthcare). MukBEF was largely found in the flowthrough and was applied to a 5 mL HiTrap Q HP column (GE Healthcare). The column was washed with 2 CV of 10 mM Tris, 200 mM NaCl, 1 mM TCEP, pH 7.4 at RT, and protein was eluted with a 20 CV linear gradient from 200 mM NaCl to 1 M NaCl in buffer Q. MukBEF eluted at about 450 mM NaCl, was concentrated to 0.5 mL on a Vivaspin 20 MWCO 30 filter and was injected into a Superose 6 Increase 10/300 GL column (GE Healthcare) in buffer H200 (20 mM HEPES, 200 mM NaCl, 1 mM TCEP, pH 7.3 at RT). Peak fractions were pooled, concentrated to 8.2 mg/mL on a Vivaspin 2 MWCO 30 filter, aliquoted, frozen in liquid nitrogen and stored at −80°C until use. Protein stoichiometry was estimated by SDS-PAGE and Coomassie staining as $MukB_2E_4F_2$–$AcpP_2$.

MukB was produced from pFB468 and purified as above except for omission of the Heparin step. Protein was concentrated to 8 mg/mL. Estimated stoichiometry was $MukB_2$–$AcpP_2$.

We were unable to establish polycistronic $MukB^{EQ}EF$ expression constructs, likely due to toxicity in the cloning host. Therefore, we cloned His_6-SUMO-$MukB^{EQ}$ and MukEF as two separate expression constructs, pFB485 and pFB486, whereby the His_6-SUMO-$MukB^{EQ}$ construct was always propagated at 22°C. Proteins were separately produced as above, with His_6-SUMO-$MukB^{EQ}$ production at 22°C. Cell pellets of both strains (15 g each) were mixed in 180 mL IMAC buffer, and the complex was purified as described above, except that 1 mM EDTA (pH 7.4 at RT) was added before application to the Heparin column with the intention to improve dissociation of potentially co-purifying nucleotides. Estimated stoichiometry was ${MukB^{EQ}}_2E_4F_2$–$AcpP_2$.

$MukB^{EQ}$ was purified as above except for omission of the Heparin step. Protein was concentrated to 8 mg/mL. Estimated stoichiometry was ${MukB^{EQ}}_2$–$AcpP_2$.

MatP

P. thracensis MatP (NCBI accession identifier WP_046976581.1) was cloned without tag (pFB469) and expressed as above. The untagged protein bound tightly to IMAC resin. Extract was prepared as above and was passed through a 5 mL HisTrap HP column (GE Healthcare). The column was washed with 10 CV of IMAC buffer, and the protein was eluted with IMAC buffer containing 270 mM imidazole. The eluate was diluted with an equal volume of buffer Q, passed through a 0.45 μm filter and applied to a 5 mL HiTrap Q HP column. MatP was largely found in the flowthrough, which was then loaded on a 5 mL HiTrap SP HP column (GE Healthcare). The column was washed with 2 CV buffer Q containing 150 mM NaCl and eluted with a 20 CV linear gradient from 150 mM to 1 M NaCl in buffer Q. The protein eluted at around 400 mM NaCl. Peak fractions were pooled, concentrated in a Vivaspin 20 MWCO 10 filter (Sartorius) to 4.8 mg/mL, aliquoted, frozen in liquid nitrogen and stored at −80°C.

matS sites and electrophoretic mobility shift assay (EMSA)

Initial binding experiments indicated that the affinity of *P. thracensis* MatP for the consensus *E. coli matS* GTGACATTGTCAC (palindrome underlined) was about an order of magnitude lower than reported for other MatP proteins (Dupaigne et al., 2012; Mercier et al., 2008). To identify alternative *matS* sites, we mapped all sites with edit distance up to 2 within the palindromic region onto the *P. thracensis* chromosome (NCBI accession identifier CP011104.1) using Wolfram Mathematica, and ranked them by median distance to the replication terminus predicted by cumulative GC skew (Lobry, 1996). This identified GTTACNNNGTAAC as an abundant site with 9 occurences (Figure S1A) which was further characterized by EMSA.

6-Carboxyfluorescein (6-FAM) labeled DNA oligonucleotides *matS1* (annealed from single stranded oligonucleotides FBA747: [6-FAM]CACTGTGACATTGTCACGGCA, and FBA748: TGCCGTGACAATGTCACAGTG; *E. coli* consensus *matS* underlined) or *matS2* (FBA765: [6-FAM]CACTGTTACAGTGTAACGGCA, and FBA766: TGCCGTTACACTGTAACAGTG; *P. thracensis* candidate site underlined) at a final concentration of 2 nM in buffer H30 (20 mM HEPES, 30 mM NaCl, 1 mM TCEP, pH 7.3 at RT) were titrated with MatP and incubated for 5 min at RT. Samples were resolved on a 6% DNA Retardation gel (Thermo Fisher Scientific) with 0.5x TBE running buffer at 100 V for 60 min at 4°C. Gels were scanned on a Typhoon TLA9000 with Cy2 setup.

Cryo-EM sample preparation

Optimal protein ratio for formation of MukBEF dimers was estimated by titrating 375 nM $MukB_2E_4F_2$–$AcpP_2$ with $MukB_2$–$AcpP_2$ in buffer H200 (20 mM HEPES, 200 mM NaCl, 1 mM TCEP, pH 7.3 at RT), followed by size exclusion chromatography on a Superose 6 Increase 3.2/300 column (GE Healthcare) mounted on an ÄKTA Ettan (GE Healthcare). An equimolar mixture shifted most protein to the dimer fraction, with some residual material in $MukB_2E_4F_2$–$AcpP_2$ and $MukB_2$–$AcpP_2$ fractions.

${MukB^{EQ}}_2E_4F_2$–$AcpP_2$ and ${MukB^{EQ}}_2$–$AcpP_2$ were mixed at 10 μM each in 4.8 μL buffer H200 (20 mM HEPES, 200 mM NaCl, 1 mM TCEP, pH 7.3 at RT) and incubated for 10 min on ice. Then, 0.48 μL of a 100 μM stock of DNA in water (annealed from oligonucleotides FBA769: CTCGCCTGTAAAGTAGGCATTAGTTGTTCGTAGTGCTCGTCTGGCTCTGGATTACCCGCCACTGTTACATTGTAACGGCA, and FBA770: TGCCGTTACAATGTAACAGTGGCGGGTAATCCAGAGCCAGACGAGCACTACGAACAACTAATGCCTACTTTACAGGCGAG; *matS* sequence underlined) and 1.8 μL of $MatP_2$ (26.8 μM stock in buffer H200) were added. After 5 min on ice, 4.9 μL of buffer H30 (20 mM HEPES, 30 mM NaCl, 1 mM TCEP, pH 7.3 at RT) were added and the sample was passed through a Zeba Micro Spin 7K MWCO column (Thermo Fisher Scientific) equilibrated in buffer H30 containing 1 mM ATP, 2 mM $MgCl_2$ and 0.05% (w/v) β-octyl glucoside. The sample was incubated for 10 min at RT, then for 30 min on ice.

Cryo-EM grids were prepared as follows. UltrAuFoil (Russo and Passmore, 2014) R2/2 200 mesh grids (Quantifoil) were glow discharged for 15 s at 30 mA in a SC7620 (Quorum), and 2.5 μL sample were applied to a freshly glow discharged grid mounted in a Vitrobot Mark IV (Thermo Fisher Scientific), equilibrated at 4°C and 100% humidity. Grids were immediately blotted at blot force −15, 2-4 s blotting time, no drain time and plunge frozen in liquid ethane. Grids were stored in liquid nitrogen until use. Sample screening and optimization was performed on TFS Tecnai F20, Polara and Glacios microscopes.

Cryo-EM data collection

Data was collected on multiple grids over three sessions on a TFS Titan Krios with X-FEG emitter at 300 kV, equipped with a Gatan K3 detector operating in counting mode and a Gatan Quantum energy filter with 20 eV slit width centered on the zero-loss peak. Datasets 1 and 2 were collected with hardware binning at 1.07 Å calibrated pixel size. Dataset 3 was acquired without hardware binning at 0.535 Å calibrated pixel size. Movies were acquired in SerialEM (Mastronarde, 2005) at three areas per hole, using image shift with beam-tilt compensation to collect 9 holes per stage movement. Target defocus was −1 to −3 μm, total electron fluence was 40 e^-/A^2 collected over 2.5 s and fractionated into 40 frames.

Genome engineering

The DNA assembly and recombination strategy is shown in Figure S4. We used the helper plasmid pKW20 (NCBI accession identifier MN927219.1) (Wang et al., 2016) for genome engineering, which constitutively expresses a tracrRNA gene, and *cas9* and λ-Red components (α, β, and γ) upon L-arabinose induction. If required, pKW20 can be efficiently cured by growth in the absence of selection (about 1 in 8 loss frequency after overnight growth and single colony plating). For construction of acceptor strains we integrated a *pheS(T251, A294G)-hyg^R* double selection cassette either downstream of the *mukFEB* operon (SFB047, SFB180) or replacing the *mukFEB* coding region (SFB053) by λ-Red recombineering (Yu et al., 2000). *pheS(T251A, A294G)* is a robust negative selection marker that encodes a mutant phenylalanyl-tRNA synthetase which confers toxicity in presence of 4-chloro-phenylalanine (4-CP) (Miyazaki, 2015). We observed that the efficiency of introducing the desired point mutations decays with distance from the selection marker and generally recommend deleting the target locus if possible. For a target locus that cannot be deleted, we recommend placing the double selection cassette at the upstream end of the locus for efficient replacement with the DNA of interest in case a downstream positive selection marker is used, or vice versa (Wang et al., 2016).

A marker-free Δ*matP* allele was constructed by replacing *matP* with *pheS(T251, A294G)-hyg^R* and subsequent cassette ejection with a double-stranded oligonucleotide coding for a *matP* in-frame deletion.

We used SFB065 as a conjugative donor; this is a DH5α strain bearing the non-transferrable conjugative plasmid pJF146 (NCBI accession identifier MK809154.1), a pRK24 derivative with a truncated nick site of the origin of transfer (*oriT*) and *apr^R* selection marker (Fredens et al., 2019). DH5α achieves high transformation efficiencies using chemical competence (Inoue et al., 1990) which aids parallelization of transformation reactions, and can be easily outcompeted by MG1655 due to its slow growth. In addition, it is *thi$^-$* and can be counter-selected on minimal media lacking thiamine.

Shuttle plasmids were constructed with a pMB1 origin of replication, a *pheS(T251, A294G)-hyg^R* cassette, an *oriT*, a CRISPR array, and a *ccdB* toxin gene flanked by *Bsa*I acceptor sites for Golden Gate assembly (Engler et al., 2008). We designed several shuttle plasmid variants: pFB448 contains a *Bsm*BI Golden Gate acceptor site for convenient assembly of custom CRISPR arrays, pFB377 contains a CRISPR array targeting *mukFEB*, pFB411 targets *mukFEB* and the pKW20 helper plasmid, and pFB449 targets *mukB* and pKW20. crRNA encoded by the CRISPR arrays were designed such that they would mediate scarless excision, using 30 bp of homology to the target (Figure S4). Plasmids were propagated in ccdB Survival cells.

The *mukFEB* locus was split into four modules, and individual modules were *Bsm*BI assembled into a backbone containing a *bla* marker (pFB017). Desired point mutations were introduced by PCR during this assembly step and modules were designed such that they could be excised and assembled with *Bsa*I (Figure S4). Module architectures as exemplified for construction of the *mukFEB* ring cysteine strain are shown in Data S1 (pFB507, pFB502, pFB508, and pFB287). If sequencing and re-use of the modules is not required (‘screening mode’), the cloning step can be omitted, and PCR products can be assembled directly into the shuttle backbone. The assembly scheme resulted in a final targeting construct that was flanked by 50 bp homology regions for recombination and protospacer adjacent motives (PAM) for Cas9 excision (Figure S4). The construct introduced a kanamycin selectable *neo^R* marker integrated downstream of the operon. Cloned modules were then *Bsa*I assembled into an appropriate shuttle backbone and transformed into SFB065 with 200 μg/mL hygromycin-B and 50 μg/mL apramycin selection.

Recipient cells were grown to stationary phase in 5 mL LB with 5 μg/mL tetracycline and 2% glucose at 37°C for *muk$^+$* (SFB047, SFB180) and at 22°C for *muk$^-$* (SFB053). If grown at 22°C, cultures were protected from light to prevent degradation of tetracycline. 4 mL were harvested by centrifugation and washed three times in 1 mL LB, transferred to 50 mL LB with 5 μg/mL tetracycline and 0.5% (w/v) L-arabinose and incubated for 1 h at 37°C. Cells were harvested by centrifugation and washed three times in 1 mL LB. Donor colonies were washed off the transformation plate using 2 mL LB. All cultures were resuspended in LB to a final OD of 40. Next, 12.5 μL recipient were mixed with 87.5 μL donor and spotted in small volumes onto a well-dried TYE plate. Spots were air-dried, and the conjugation plate was incubated at 30°C for 1 h. Cells were washed off and transferred into 50 mL LB with 12.5 μg/mL kanamycin and 5 μg/mL tetracycline. In cases where pKW20 was targeted by the CRISPR array, tetracycline was omitted. If the conjugated construct was *muk$^+$*, cells were incubated for 4 h at 37°C to allow for recombination and loss of the counter-selection marker. If the construct was *muk$^-$*, cells were incubated for 1 h at 37°C and then overnight at 22°C. Serial dilutions were plated and colonies were selected on LB with 12.5 μg/mL kanamycin and 2.5 mM 4-CP, and additional 5 μg/mL tetracycline and 2% glucose if pKW20 was to be maintained. In some cases, cells were selected on M9 glucose with 50 μg/mL kanamycin and 2.5 mM 4-CP, without thiamine.

All strains were single colony purified, and verified by marker analysis, phenotype, PCR, and Sanger sequencing as required. Annotated shuttle plasmid sequences, *mukFEB* GoldenGate module architecture, and architecture of genomic loci are provided in Data S1. Plasmids are listed in Table S2.

In vivo cross-linking

Cells were grown to exponential phase in LB (OD 0.2-0.3) if not indicated otherwise. Cultures were mixed with 30% (w/v) ice, harvested by centrifugation, and kept cold for the duration of the experiment. 0.5 OD units of cells were washed in 500 μL of ice-cold PBS and resuspended in 50 μL PBS. Next, 1.25 μL of BMOE (20 mM stock in DMSO) were added and the suspension was incubated for 10 min on ice. The reaction was quenched by addition of 1 μL of 2-mercaptoethanol (2-ME, 1.4 M stock in water). Cells were resuspended in 50 μL of B-PER (Thermo Fisher Scientific) containing 1 mM EDTA (pH 7.4 at RT), 14 mM 2-ME, protease inhibitor cocktail (Roche), 1 μM HaloTag-TMR substrate (Promega), 0.25 U/μL Benzonase (Merck), and 0.1 U/μL ReadyLyse Lysozyme

(Lucigen). The suspension was incubated for 5 min at RT and then for 10 min at 37°C, after which 16.6 μL of 4x LDS sample buffer (Thermo Fisher Scientific) containing 6% (v/v) 2-ME were added and the sample was incubated for 5 min at 95°C. 10 μL of sample were resolved on NuPAGE 4%–12% Bis-Tris gels (Thermo Fisher Scientific) using MOPS running buffer. Gels were scanned on a Typhoon FLA9000 (GE Healthcare) with Cy3 setup.

Chromosome entrapment assay

The *E. coli* chromosome entrapment assay was based on protocols developed for *B. subtilis* (Vazquez Nunez et al., 2019; Wilhelm et al., 2015). Stationary phase cultures were inoculated into 100 mL LB and grown to OD 0.2-0.3 at 22°C, which is the permissive temperature for ATPase deficient *mukFEB* strains. We obtained similar results at 37°C for WT ATPase strains. Cells were harvested by centrifugation, washed in 1 mL of ice-cold PBS, and 14 OD units were resuspended in 720 μL PBS. 18 μL BMOE (20 mM in DMSO) were added, and the suspension was incubated for 10 min on ice. The reaction was quenched by addition of 14.4 μL 2-ME (1.4 M in water). Cells were resuspended in 200 μL PBS containing 10 mM EDTA (pH 7.4 at RT), protease inhibitor cocktail (Roche), and 5 μM HaloTag-TMR substrate (Promega) and incubated for 15 min at 37°C with shaking. Samples were protected from light from now on. For input samples, 0.5 OD units from the labeling mix were collected, resuspended in 50 μL B-PER (Thermo Fisher Scientific) with 1 mM EDTA (pH 7.4 at RT), protease inhibitor cocktail, 0.25 U/μL Benzonase (Merck), and 10 U/μL ReadyLyse lysozyme (Lucigen) and incubated for 1 h at RT. Input samples were stored at −20°C after addition of 16.6 μL 4x LDS sample buffer containing 6% (v/v) 2-ME. Two agarose plugs per sample were formed each by mixing 100 μL cells with 100 μL low-melt agarose (2% (w/v), freshly melted at 80°C and equilibrated to 45°C) in the bottom of a 2 mL tube, and incubation of the suspension for 5 min on ice. Both plugs were pooled into 3 mL B-PER with 10 mM EDTA (pH 7.4 at RT), protease inhibitor cocktail, and 10 U/μL ReadyLyse lysozyme and incubated for 2.5 h at RT on a roller. Plugs were transferred into 50 mL TGES (25 mM Tris, 192 mM glycine, 10 mM EDTA (pH 7.4 at RT), 0.1% SDS) and incubated for 2 h at RT on a roller. Plugs were mounted in a 1% (w/v) agarose gel in TGES and subjected to electrophoresis in TGES at 10 mA/cm^2 for 1.5 h in a chamber cooled on ice. Next, plugs were transferred into 50 mL PBS, incubated for 2 h at RT on a roller, then transferred to 2 mL tubes and melted at 80°C for 3 min with occasional vortexing. The solution was incubated at 45°C for 5 min, thoroughly mixed with 200 μL PBS containing 20 mM $MgCl_2$ and 0.25 U/μL Benzonase and solidified on ice. Samples were incubated at 37°C for 30 min and stored at −80°C overnight. Next, samples were thawed at RT for 20 min and spun at 21,000 x g and 4°C for 15 min. Extract from both plugs was combined and passed through a 0.45 μm Costar Spin-X filter (Corning) by centrifugation at 10,000 x g for 1 min. Samples were brought to 1 mL with water before addition of 6 μL BSA (1 mg/mL) and 110 μL of 100% (w/v) trichloroacetic acid. Tubes were incubated on ice for 30 min and precipitated protein was collected by centrifugation at 21,000 x g and 4°C for 15 min, careful removal of the supernatant, and a second spin for 3 min to remove remaining liquid. The precipitate, which formed a haze on the tube wall, was dissolved in 20 μL 2x LDS sample buffer containing 3% (v/v) 2-ME. Samples were incubated for 5 min at 95°C, and 5 μL of input and 10 μL of eluate were separated on NuPAGE 3%–8% Tris-Acetate gels (Thermo Fisher Scientific) run at 4°C and 35 mA/gel for 1.5 h. Gels were scanned on a Typhoon FLA9000 (GE Healthcare) with Cy3 setup.

QUANTIFICATION AND STATISTICAL ANALYSIS

Cryo-EM data analysis

An overview of the data analysis workflow is shown in Figure S2. Motion correction and dose weighting was performed in RELION (Scheres, 2012) with one patch per micrograph and on-the-fly gain correction. Super-resolution data of dataset 3 were binned by a factor of 2. The contrast transfer function (CTF) was fitted with CTFFIND4 (Rohou and Grigorieff, 2015). Automated particle picking was performed with crYOLO (Wagner et al., 2019). All further processing was done in RELION.

An initial model of MukBEF was reconstructed from an exploratory dataset collected on apo-$MukB_2E_4F_2$–$AcpP_2$ (Figure S2A). Particles were picked with a custom trained crYOLO model and subjected to 2D classification. Particles from good classes were used for *ab initio* reconstruction. The model was manually sculpted at to remove obvious artifacts, and iteratively improved by 3D classification, auto-refinement, sculpting, optimization of the crYOLO model and repicking.

$MukB^{EQ}$EF–MatP–DNA particles were picked from dataset 1 using the apo-MukBEF crYOLO model. Using the apo-MukBEF reconstruction as a starting model, an initial model for $MukB^{EQ}$EF–MatP–DNA was obtained after rounds of refinement, 2D classification without alignment, 3D classification with global pose search and masked 3D classification without alignment focused on the joint (Figure S2A). Next, particles from all datasets were extracted at 4.3 Å/px and partitioned into optics groups by hole position and grid. The initial model was separately refined against batches of particles, and batches were cleaned by 2D classification without alignment. Particles were re-extracted with re-centering at 4.3 Å/px, and the initial model was refined against all of them. Particles were then subjected to 3D classification without alignment. The best class showed residual density for additional monomers arranged in a tetrad (Figures 3C and S2A). Monomers of the tetrad were extracted at 1.45 Å/px and pooled. Refinement with global pose search, followed by duplicate removal and refinement with local pose search resulted in a map at 6.2 Å resolution. This was further classified without alignment using a mask around the arms, followed by signal subtraction of the head module, and focused refinement and 3D classification without alignment of the elbow region. After reverting to original particles, focused refinement of the head module followed by CTF refinement (beam tilt, anisotropic magnification, per-particle defocus) and Bayesian polishing were performed. A final refinement that contained the full complex inside its mask yielded a map at an overall nominal resolution of 4.6 Å.

CellPress
OPEN ACCESS

Reconstructions of the open states and apo complex as shown in Figure 4A were obtained by branching off the main processing tree. Similarly, low resolution reconstructions for the apo dimer and DNA-bound tetrads (Figures 3B–3D) were obtained by re-centering and sub-classification of classes from the main tree.

A focused reconstruction of the MukBEF–MatP–DNA head module was obtained as follows. Datasets were processed separately as shown in Figure S2B. Briefly, head modules from monomers within a tetrad were extracted with re-centering, subjected to multiple rounds of 3D classification, 3D refinement, CTF refinement (beam tilt, anisotropic magnification, per-particle defocus) and a final round of Bayesian polishing. Duplicate removal was performed on multiple occasions to exclude the same head in a tetrad contributing multiple times. At final stages, refinement was performed with local pose search. Using a mask around the head module, this resulted in two maps, one from pooled datasets 1 and 2 at 3.5 Å resolution, and one from dataset 3 at 3.4 Å resolution. After pooling particles, the combined refinement yielded a map at 3.3 Å resolution. Re-centering on the holocomplex with box expansion, followed by CTF refinement and Bayesian polishing yielded a final map at 3.1 Å resolution.

Maps were rendered in ChimeraX (Pettersen et al., 2021). Fourier shell correlation (FSC) for half-maps was computed in PHENIX and is shown in Figure S2C. Data collection and map statistics are shown in Table 1.

Structural model building

First, a model for the head module was built into its map at 3.1 Å resolution, sharpened by B-factor compensation and FSC weighting (Rosenthal and Henderson, 2003) in RELION PostProcess. Homology models were obtained from PDB entries 3EUJ, 3EUH, 3VEA and 3IBP (Dupaigne et al., 2012; Li et al., 2010; Woo et al., 2009) using SWISS-MODEL (Waterhouse et al., 2018), and rigid body fitted using ChimeraX. PDB: 6DFL was used as a starting model for AcpP (Kreamer et al., 2018). The hinge-proximal arm region was flexibly fitted by interactive molecular dynamics simulation in ISOLDE (Croll, 2018). The model was then partially rebuilt in Coot (Emsley et al., 2010), whereby the necks, joints and parts of the hinge-proximal arm were built *de novo*. The clamped DNA was modeled as poly-AT. Next, the model was subjected to phenix.real_space_refine (Afonine et al., 2018) to resolve major clashes, and annealed using ISOLDE. The model was improved by cycles of editing in ISOLDE and Coot, and automated refinement in phenix.real_space_refine using secondary structure restraints (alpha, beta, base-pair), Ramachandran restraints, without non-crystallographic symmetry (NCS) constraints. Model versus map FSC was computed in PHENIX and is shown in Figure S2C.

A medium resolution model for the complete MukBEF–MatP–DNA monomer was built as follows. The head module was rigid body fitted into the 4.6-Å holocomplex map sharpened in RELION PostProcess. Homology models for hinge and elbow were obtained from PDB: 3IBP and 6H2X (Bürmann et al., 2019; Li et al., 2010), respectively, using SWISS-MODEL. Homology models were split and flexibly fitted using ISOLDE. Connecting segments between head- and hinge-proximal arms and the elbow coiled coil were built *de novo*. There was little or no sidechain information for these segments, but their simple coiled-coil architecture and highly constrained ends allowed building of a realistic model. For example, residues of the predicted hydrophobic heptad-repeat patterns locate to the helix interfaces, and prolines locate to helix breaks. The model was improved by editing in ISOLDE and Coot, and automated refinement in phenix.real_space_refine using reference model restraints for hinge and head module, secondary structure restraints, Ramachandran restraints, and no NCS constraints. Model versus map FSC was computed in PHENIX and is shown in Figure S2C.

Models for apo state and states with more open arm conformations were obtained by flexible fitting of the holocomplex reference model in ISOLDE followed by automated refinement in phenix.real_space_refine.

Low resolution models for dimers and tetrads were obtained by rigid body fitting of holocomplex monomers into multimer maps blurred to 20 Å resolution. Connecting peptides in MukF were built in Coot with a homology model of the MukF dimer based on PDB entry 3EUH (Woo et al., 2009) as a guide. The models contained some clashes at the MukF interfaces, some of which were resolved by changing sidechain rotamers without touching the main chain. Next, idealized 80-bp double-stranded DNA was generated in Coot and flexibly fitted in ISOLDE using strong distance restraints. Protein-bound regions of the DNA were replaced by the corresponding parts of the rigid-body docked medium resolution DNA models. The head-bound poly-AT models were edited to match the oligonucleotide sequence. DNA was subjected to a single cycle of phenix.real_space_refine with base pair and input model restraints to resolve major geometry errors. For the tetrad with dimers directly apposed (Figure 3D), only two monomers were modeled due to weak density for the remaining two monomers.

Model Figures were rendered in ChimeraX. Movies were rendered in PyMOL (Schrödinger). Model statistics are listed in Table 1.

Analysis of EMSA experiments

Quantification of bands was performed with Wolfram Mathematica using moving median estimation of background signal. Data were fit by a rate equation model at equilibrium in an arbitrary time domain, parametrized by dissociation rate k_d, baseline and asymptote, and with the association rate k_a as an arbitrary model constant. The equilibrium dissociation constant K_d was determined as k_d/k_a which is independent of the time domain. This approach is easily extendable to reaction models where analytical solutions or approximations are difficult to derive and can be adapted to time resolved detection methods for determination of true rate constants.

Analysis of cross-linking experiments

Quantification of bands was performed with Wolfram Mathematica using moving median estimation of background signal. Credible intervals were estimated from posterior distributions using a normally distributed likelihood with mean μ and standard deviation σ, a uniform prior over [0, 1] for μ and a $1/\sigma^2$ prior for σ.

Analysis of chromosome entrapment assays

Quantification of bands was performed with Wolfram Mathematica using moving median estimation of background signal. Credible intervals were estimated from posterior distributions using a normally distributed likelihood with mean μ and standard deviation σ, a uniform prior over [-10 μ, 10 μ] for μ and a $1/\sigma^2$ prior for σ.

Molecular Cell, Volume *81*

Supplemental information

Cryo-EM structure of MukBEF reveals DNA loop entrapment at chromosomal unloading sites

Frank Bürmann, Louise F.H. Funke, Jason W. Chin, and Jan Löwe

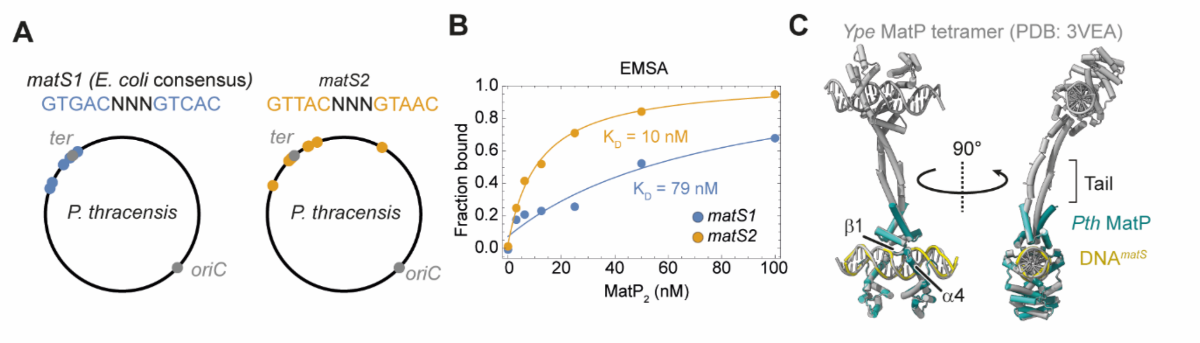

Figure S1. *matS* binding by *P. thracensis* MatP. Related to **Figure 1**.
(**A**) Location of the *E. coli matS* consensus sequence (left) and the *matS* sequence used for structure determination (right) mapped onto the *P. thracensis* chromosome. (**B**) Affinities of *P. thracensis* MatP for *matS* sites shown in A as determined by EMSA. (**C**) Superimposition of MatP–*matS* in the MukBEF-bound form (colored) and a crystal structure in the absence of MukBEF (gray, PDB: 3VEA). Positions of the *matS* binding elements α4 and β1 and the C-terminal tetramerization tail are indicated. *Ype*, *Yersinia pestis*; *Pth*, *P. thracensis*.

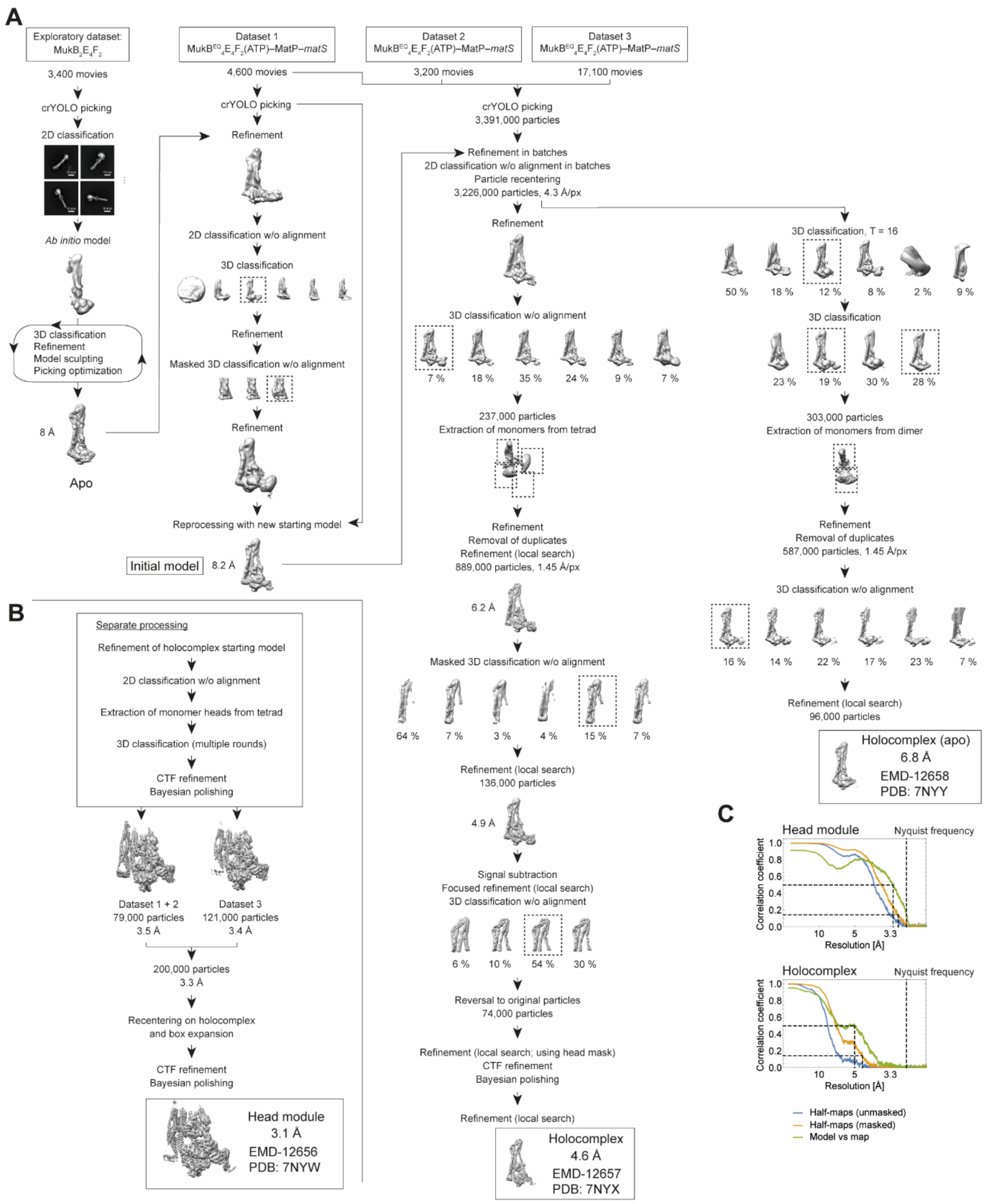

Figure S2. Cryo-EM data analysis workflow. Related to **Figures 1**-**3**.
(**A**) The processing tree for structure determination of MukBEF–MatP–DNA and apo-MukBEF monomers. (**B**) Data processing tree for focused structure determination of the head module. (**C**) FSC curves for MukBEF–MatP–DNA head module and holocomplex structures.

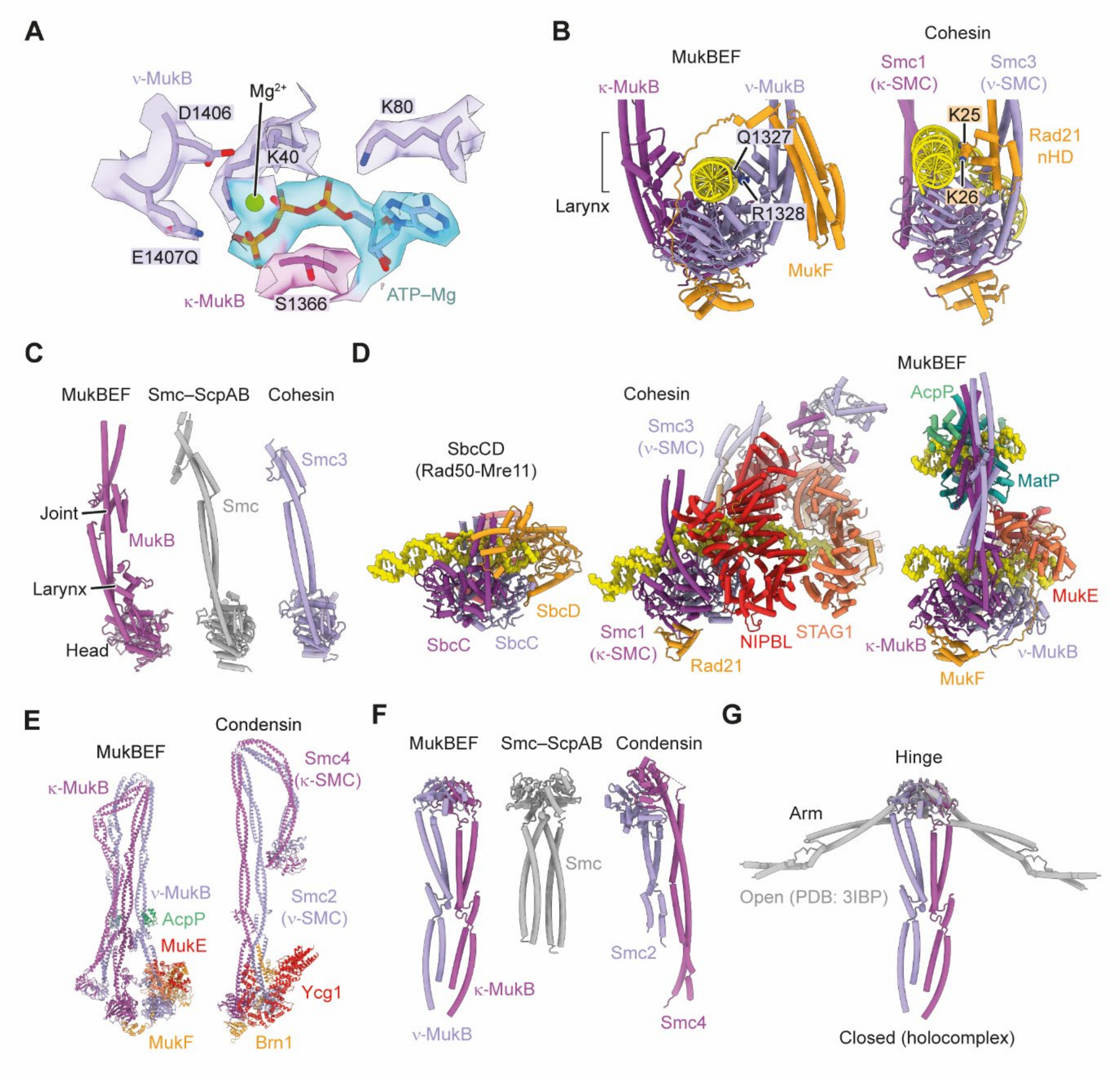

Figure S3. Conserved structural features and comparison with other SMC complexes. Related to **Figures 1**-**4**.

(**A**) Cryo-EM density and atomic model for the nucleotide binding site at the ν-MukB Walker A and B motives and the κ-MukB signature motive. (**B**) Architecture of the neck gate in MukBEF (left) and cohesin (right, PDB: 6WG3). An asymmetric DNA contact at the larynx of MukBEF and the N-terminal helical domain of Rad21 is indicated. (**C**) Comparison of the head-proximal regions of MukB, Smc (PDB: 5XEI) and Smc3 (PDB: 6WGE). (**D**) Architecture of SMC–DNA clamps. Models were aligned on the κ-SMC ATPase domain. PDB: 6S85, 6WG3.
(**E**) Comparison of apo-MukBEF and apo-condensin (PDB: 6YVU). (**F**) Comparison of the hinge-proximal regions of MukB, Smc (PDB: 4RSJ) and Smc2/4 (PDB: 4RSI).
(**G**) Superimposition of the hinge-proximal region of MukB in an open conformation (PDB: 3IBP) and in the closed conformation.

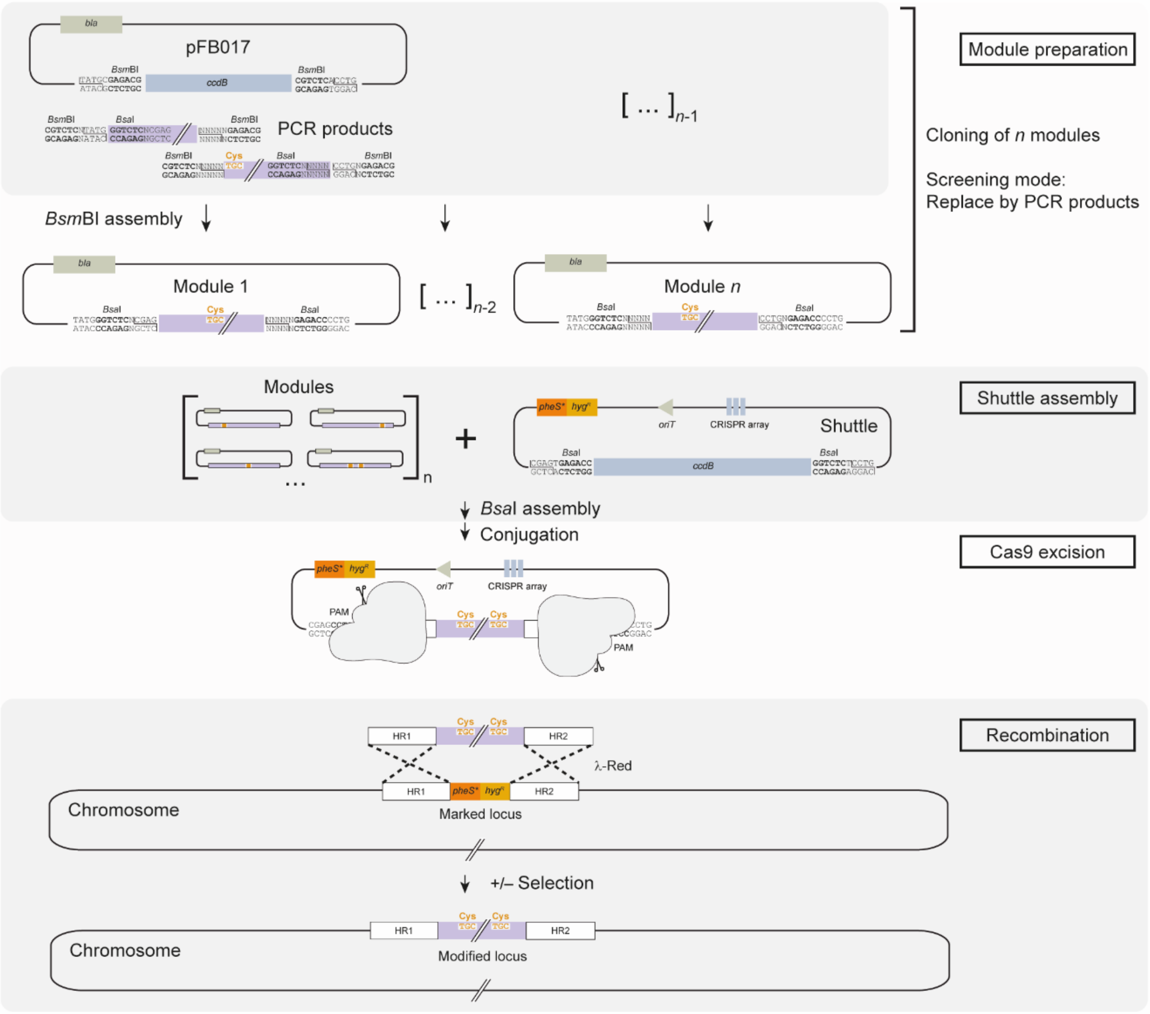

Figure S4. DNA assembly and excision scheme used for REXER-based strain construction. Related to **Figure 5**.

Modules containing cysteine point mutations were prepared as cloned and verified plasmids but can be substituted by linear PCR products (module preparation phase). Modules were assembled into a shuttle backbone (shuttle assembly phase), conjugated, and excised *in vivo* (Cas9 excision phase). Design of 30 bp homology crRNA for scarless Cas9 excision is indicated. The targeting construct is designed such that 50 bp homology regions (HR) direct the recombination into the target locus (recombination phase). Recombinants are selected for a positive marker in the targeting construct and against the *pheS** marker at the recipient locus.

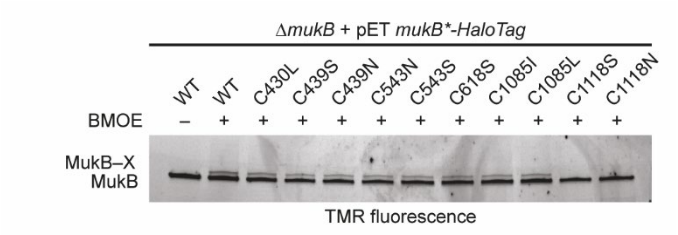

Figure S5. Background cross-linking of endogenous MukB cysteines. Related to **Figure 5**. Screening of endogenous cysteines for background cross-linking. *E. coli* Δ*mukB* was transformed with plasmids carrying *mukB-HaloTag* variants under control of a T7 promoter. Leaky expression in the strain lacking a T7 RNA polymerase gene produced about 40 **%** MukB-HaloTag compared to *mukB-HaloTag* expressed from the endogenous locus. The *mukB* null phenotype was complemented in all cases. Cells were treated with BMOE and proteins were detected by in-gel fluorescence. C1118S and C1118N abolished background cross-linking with a low-molecular weight protein.

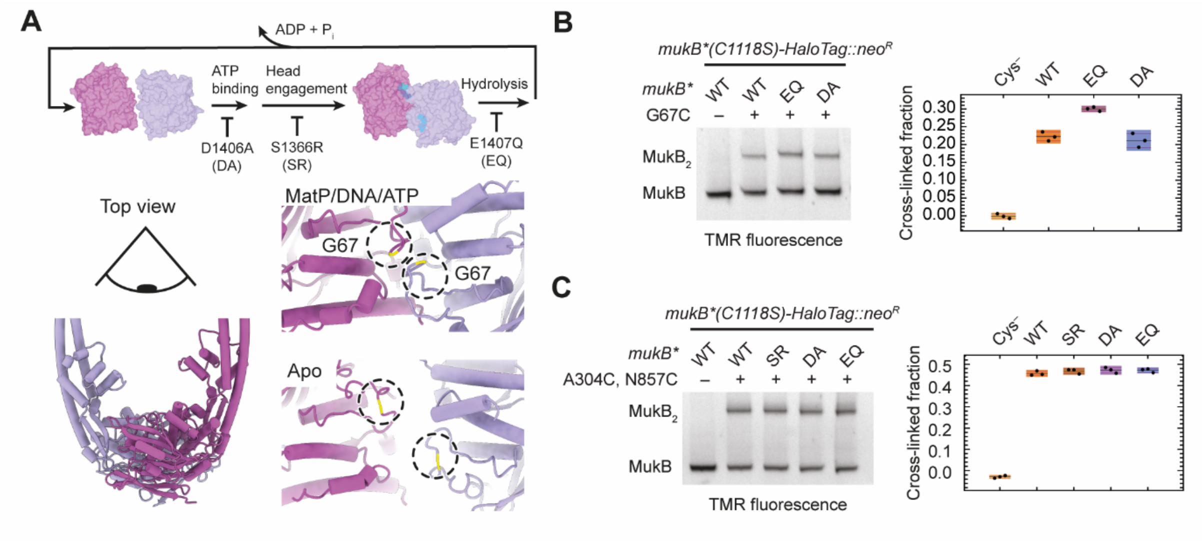

Figure S6. *In vivo* head engagement and arm folding in ATPase mutants. Related to **Figures 5** and **6**.

(**A**) Schematic of the ATP hydrolysis cycle and blocking mutations (top). Location of the head engagement sensor residue G67 in MatP/DNA/ATP and apo states (bottom). S1366R ('SR') blocks head engagement, D1406A ('DA') blocks ATP binding, E1407Q ('EQ') blocks ATP hydrolysis. (**B**) BMOE cross-linking of strains carrying the head engagement sensor mutation G67C and ATPase blocking mutations. Cells were grown in LB for 1.5 h at 37 °C before cross-linking. In-gel fluorescence is shown on the left, and quantification of three technical replicates is shown on the right. Black lines indicate means, purple lines indicate standard deviations, and colored bars indicate 95 % credible intervals. (**C**) BMOE cross-linking of arm folding sensor strains carrying ATPase mutations. As in B.

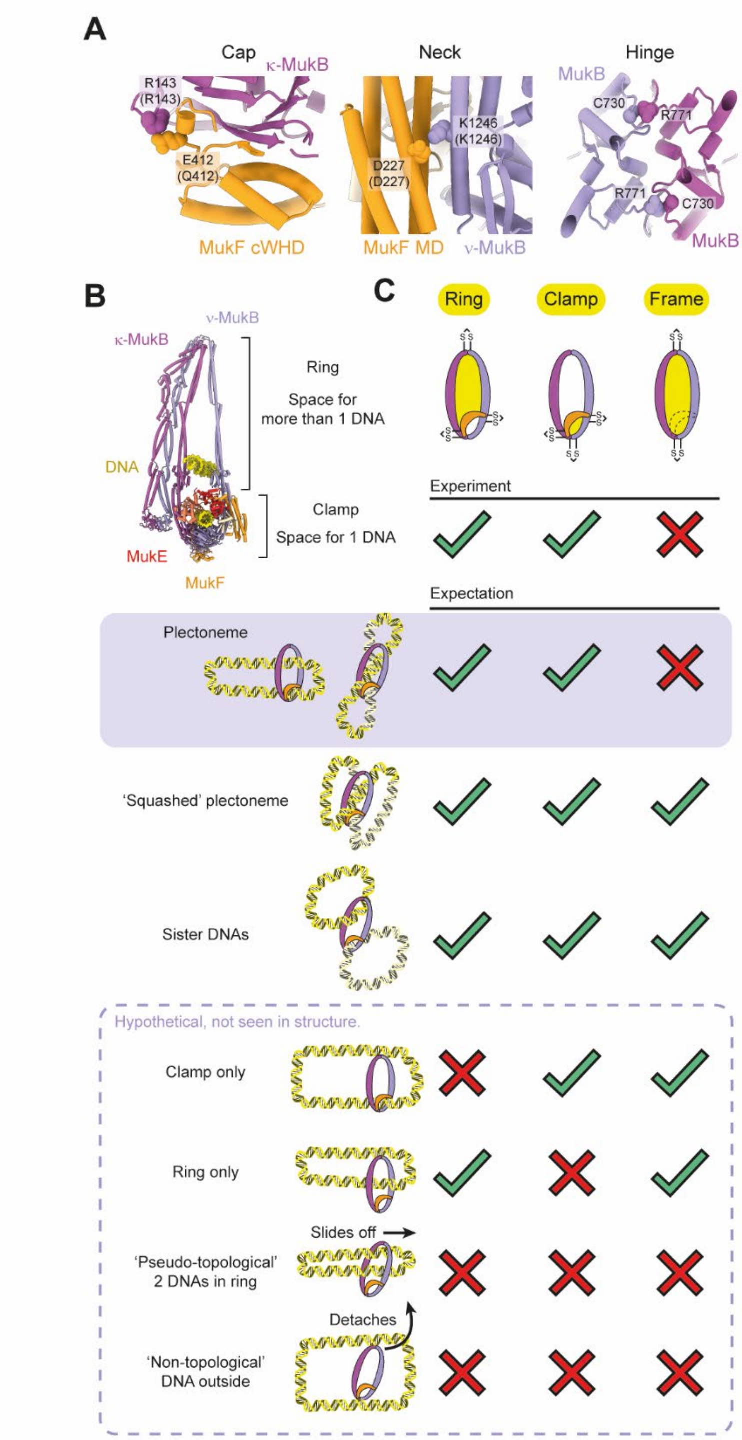

Figure S7. Chromosome entrapment by MukBEF. Related to **Figure 6**.
(**A**) Design of cap, neck and hinge cross-links. Location of residues chosen for cysteine mutagenesis at the hinge (left, PDB: 3IBP), cap (middle), and neck (right) is shown. Labels for cap and neck are shown for the *P. thracensis* structure and corresponding *E. coli* residues are in parentheses. (**B**) Space for hypothetical accommodation of additional DNA double strands. Only the ring compartment is large enough to embrace more than one DNA.
(**C**) Comparison of experimentally observed and expected catenanes for different DNA binding topologies. The topology consistent with the experimental data is highlighted. For clarity, the double-locked plectoneme topology is also shown as a simplified version without

DNA crossings. Hypothetical ‘pseudo-topological’ and ‘non-topological’ DNA loops do not produce catenanes. Their tentative formation in addition to catenated forms is therefore not excluded by the data.

Table S1. Bacterial strains. Related to STAR Methods.

Strain ID	Genotype	Figures
BL21-Gold(DE3)	F-, *lon-*, *ompT-*, *hsdS*(*rb- mb-*), *dcm+*, *tet*, *gal*, λ(DE3), *endA-*, *Hte*	
C41(DE3)	F-, *ompT, gal, dcm, hsdSB*(*rB- mB-*), λ(DE3)	
MG1655	F-, λ-, *rph*-1, *fnr+*	
SFB018	MG1655, Δ*mukB::neoR*	S5
SFB047	MG1655, *mukB::pheS(T251A, A294G)-hygR*, pKW20 Para *lambda-red cas9 tet tracrRNA*	
SFB053	MG1655, Δ*mukFEB::pheS(T251A, A294G)-hygR*, pKW20 Para *lambda-red cas9 tet tracrRNA*	
SFB065	DH5α, pJF146 RK24 *lux apR bsd*	
SFB114	MG1655, *mukB(A304C, N857C, C1118S)-TEV-HaloTag(C61V, C262A)::neoR*	5B, S6C
SFB115	MG1655, *mukB(G67C, C1118S)-TEV-HaloTag(C61V, C262A)::neoR*	6C, S6B
SFB116	MG1655, *mukB(C1118S)-TEV-HaloTag(C61V, C262A)::neoR*	5B, S6B, S6C
SFB117	MG1655, *mukB(A304C, C1118S)-TEV-HaloTag(C61V, C262A)::neoR*	5B
SFB118	MG1655, *mukB(N857C, C1118S)-TEV-HaloTag(C61V, C262A)::neoR*	5B
SFB119	MG1655, *mukB(R771C, C1118S)-TEV-HaloTag(C61V, C262A)::neoR*	6C
SFB120	MG1655, *mukB(G67C, C1118S, E1407Q)-TEV-HaloTag(C61V, C262A)::neoR*	S6B
SFB121	MG1655, *mukB(C730S, R771C, C1118S)-TEV-HaloTag(C61V, C262A)::neoR*	6C
SFB122	MG1655, *mukB(G67C, C1118S, D1406A)-TEV-HaloTag(C61V, C262A)::neoR*	S6B
SFB126	MG1655, *mukB(A304C, N857C, C1118S, S1366R)-TEV-HaloTag(C61V, C262A)::neoR*	S6C
SFB127	MG1655, *mukB(A304C, N857C, C1118S, D1406A)-TEV-HaloTag(C61V, C262A)::neoR*	S6C
SFB128	MG1655, *mukB(A304C, N857C, C1118S, E1407Q)-TEV-HaloTag(C61V, C262A)::neoR*	S6C
SFB167	MG1655, *mukF(D227C) mukE mukB(C1118S)-TEV-HaloTag(C61V, C262A)::neoR*	6C
SFB168	MG1655, *mukB(C1118S, K1246C)-TEV-HaloTag(C61V, C262A)::neoR*	6C
SFB169	MG1655, mukF(D227C) mukE mukB(C1118S, K1246C)-TEV-HaloTag(C61V, C262A)::neoR	6C
SFB170	MG1655, *mukF(Q412C) mukE mukB(C1118S)-TEV-HaloTag(C61V, C262A)::neoR*	6C
SFB171	MG1655, *mukF(Q412C) mukE mukB(R143C, C1118S)-TEV-HaloTag(C61V, C262A)::neoR*	6C
SFB172	MG1655, *mukF(Q412C) mukE mukB(R143C, R771C, C1118S)-TEV-HaloTag(C61V, C262A)::neoR*	6D, 6C
SFB173	MG1655, *mukF(D227C, Q412C) mukE mukB(R143C, C1118S, K1246C)-TEV-HaloTag(C61V, C262A)::neoR*	6D, 6C
SFB174	MG1655, *mukF(D227C, Q412C) mukE mukB(R143C, R771C, C1118S, K1246C)-TEV-HaloTag(C61V, C262A)::neoR*	6C-F, 6H
SFB180	MG1655, *mukF(D227C, Q412C) mukE mukB(R143C, R771C, C1118S, K1246C)::pheS(T251A, A294G)-hygR*, pKW20 Para *lambda-red cas9 tet tracrRNA*	
SFB183	MG1655, *mukB(R143C, C1118S)-TEV-HaloTag(C61V, C262A)::neoR*	6C
SFB184	MG1655, *mukF(D227C) mukE mukB(R771C, C1118S, K1246C)-TEV-HaloTag(C61V, C262A)::neoR*	6H, 6C
SFB188	MG1655, *mukB(G67C, R771C, C1118S)-TEV-HaloTag(C61V, C262A)::neoR*	6C, 6F
SFB190	MG1655, *mukF(D227C, Q412C) mukE mukB(R143C, R771C, C1118S, K1246C, D1406A)-TEV-HaloTag(C61V, C262A)::neoR*	6D, 6H
SFB191	MG1655, *mukF(D227C, Q412C) mukE mukB(R143C, R771C, C1118S, K1246C, E1407Q)-TEV-HaloTag(C61V, C262A)::neoR*	6D
SFB192	MG1655, *mukB(G67C, R771C, C1118S, D1406A)-TEV-HaloTag(C61V, C262A)::neoR*	6F
SFB193	MG1655, *mukB(G67C, R771C, C1118S, E1407Q)-TEV-HaloTag(C61V, C262A)::neoR*	6F
SFB202	MG1655, Δ*matP*, *mukF(D227C, Q412C) mukE mukB(R143C, R771C, C1118S, K1246C)-TEV-HaloTag(C61V, C262A)::neoR*	6H
SFB203	MG1655, *mukF(D227C, Q412C) mukE mukB(G67C, R143C, C1118S, K1246C)-TEV-HaloTag(C61V, C262A)::neoR*	6C, 6E
SFB204	MG1655, *mukF(D227C, Q412C) mukE mukB(G67C, R143C, C1118S, K1246C, D1406A)-TEV-HaloTag(C61V, C262A)::neoR*	6E
SFB205	MG1655, *mukF(D227C, Q412C) mukE mukB(G67C, R143C, C1118S, K1246C, E1407Q)-TEV-HaloTag(C61V, C262A)::neoR*	6E
SFB206	MG1655, *mukF(D227C, Q412C) mukE mukB(G67C, R143C, C1118S)-TEV-HaloTag(C61V, C262A)::neoR*	6C
SFB207	MG1655, *mukF(D227C, Q412C) mukE mukB(G67C, C1118S, K1246C)-TEV-HaloTag(C61V, C262A)::neoR*	6C

Table S2. Plasmids. Related to STAR Methods.

ID	Name	Description	Source
pFB017	pET-Gold1 *ccdB*	GoldenGate *BsmB*I acceptor plasmid	This study
pFB083	pGEX GST-hSENP1	T7 expression plasmid for producing GST-tagged hSENP1	Komander lab
pFB287	pET-Gold1 'mukB-TEV-HaloTag(C61V, C262A) *neoR*	Module for targeting of the *mukFEB* locus (*Bsa*I donor)	This study
pFB377	pCONEX-Gate4 CRISPR(mukFEB) *ccdB*	Shuttle plasmid for targeting of the *mukFEB* locus (*Bsa*I acceptor)	This study
pFB403	pET-Gate2 *Pth* MukF MukE His6-SUMO-MukB	T7 expression plasmid for producing SUMO-tagged MukBEF	This study
pFB411	pCONEX-Gate4 CRISPR(mukFEB cloDF13) *ccdB*	Shuttle plasmid for targeting of the *mukFEB* locus (*Bsa*I acceptor); crRNA targets pKW20 plasmid	This study
pFB448	pCONEX-Gate4 BsmBI *ccdB*	Shuttle plasmid for conjugative gene targeting; *BsmB*I acceptor site for inserting custom spacers	This study
pFB449	pCONEX-Gate5 CRISPR(mukB cloDF13) *ccdB*	Shuttle plasmid for targeting of *mukB* (*Bsa*I acceptor)	This study
pFB468	pET-Gate2 *Pth* His6-SUMO-MukB	T7 expression plasmid for producing MukB	This study
pFB469	pET-Gold1 *Pth* MatP	T7 expression plasmid for producing MatP	This study
pFB485	pET-Gate2 *Pth* His6-SUMO-MukB(E1407Q)	T7 expression plasmid for producing SUMO-tagged MukB(E1407Q)	This study
pFB486	pET-Gate2 *Pth* MukFE	T7 expression plasmid for producing MukFE	This study
pFB502	pET-Gold1 (3-723)mukB(R143C)	Module for targeting of the *mukFEB* locus (*Bsa*I donor)	This study
pFB507	pET-Gold1 mukF(D227C, Q412C)EB'	Module for targeting of the *mukFEB* locus (*Bsa*I donor)	This study
pFB508	pET-Gold1 (723-1480)mukB(R771C, C1118S, K1246C)	Module for targeting of the *mukFEB* locus (*Bsa*I donor)	This study
pJF146	RK24 *lux apR bsd*	RK2 conjugation machinery; NCBI: MK809154.1	Fredens et al., 2019
pKW20	Para *lambda-red cas9 tet tracrRNA*	REXER helper plasmid; NCBI: MN927219.1	Wang et al., 2016
